# Supplementary material for: Antitumor effects of chemically modified miR-143 lipoplexes in a mouse model of pelvic colorectal cancer via myristoylated alanine-rich C kinase substrate downregulation
Source: Mol Ther Nucleic Acids. 2023 Nov 15;34:102079. doi: 10.1016/j.omtn.2023.102079 (PMC10783569; doi:10.1016/j.omtn.2023.102079)
Supplement: Document S1. Figures S1–S4 and Table S1 [file mmc1.pdf]

OMTN, Volume *34*

Supplemental information

Antitumor effects of chemically modified miR-143 lipoplexes in a mouse model of pelvic colorectal cancer via myristoylated alanine-rich C kinase substrate downregulation

Jun Arima, Kohei Taniguchi, Nobuhiko Sugito, Kazuki Heishima, Yoshihisa Tokumaru, Yosuke Inomata, Kazumasa Komura, Tomohito Tanaka, Masa-Aki Shibata, Sang-Woong Lee, and Yukihiro Akao

Figure S1

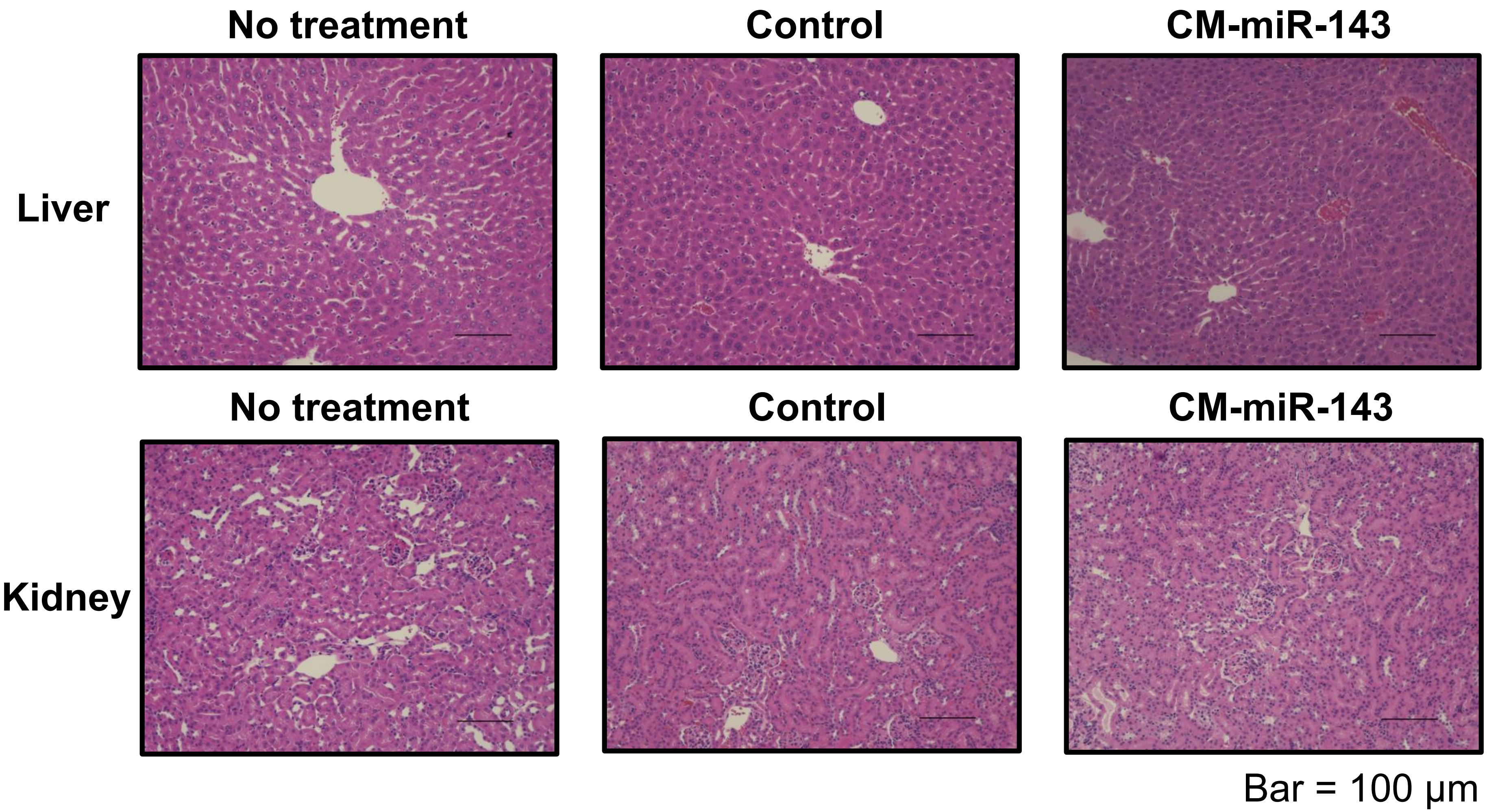

Figure S1. Representative images of pathological liver and kidney.

Liver and kidney images in no treatment, control, and CM-miR-143 groups. In control and CM-miR-143 groups, miRNA lipoplexes were administered at least six times before euthanization. No apparent differences between the liver and kidney were detected across the three groups. No treatment group: mice neither administered miRNA nor Invivofectamine™ 3.0.

Figure S2

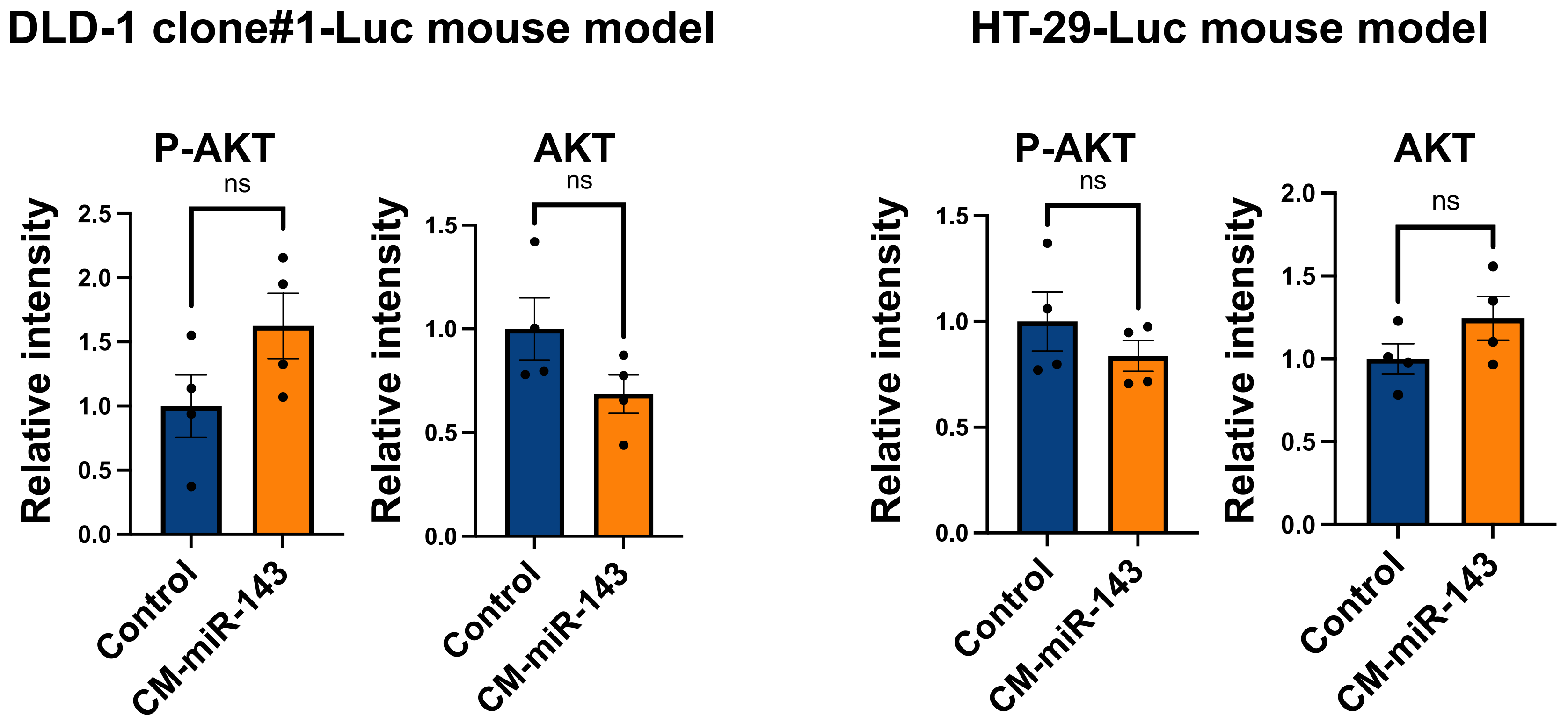

Figure S2. Quantitative intensity of P-AKT and AKT bands.

Quantitative intensity of P-AKT and AKT bands from western blots (Figure 3E) were calculated in ImageJ. Neither protein differed significantly between the control and CM-miR143 groups, based on tumor samples from DLD-1 clone#1-Luc and HT-29-Luc mouse models. Data are presented as mean ± SEM (*P < 0.05; n = 4). SEM, standard error of the mean.

Figure S3

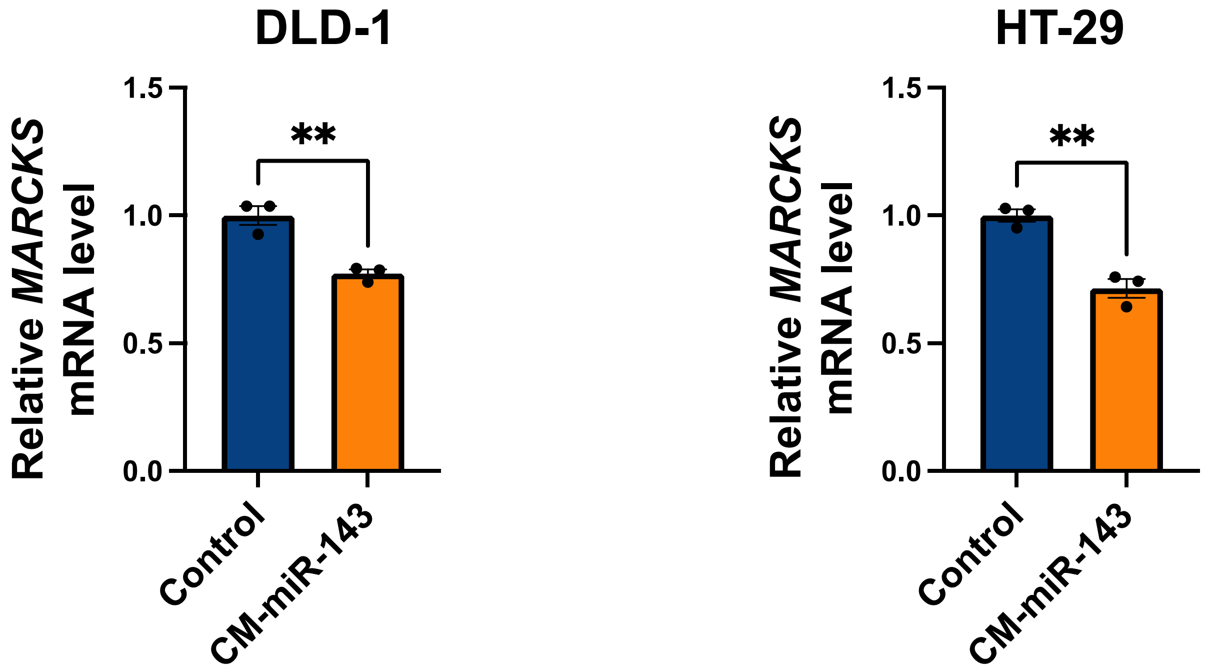

Figure S3. *MARCKS* mRNA expression level

The level of *MARCKS* mRNA expression was evaluated by qRT-PCR 48 h after transfection with control (10 nM) or CM-miR-143 (10 nM) with lipofectamine RNAiMAX in DLD-1 or HT-29 cells. CM-miR-143 suppressed *MARCKS* mRNA expression compared with the control. *GAPDH* was used as an internal control. Data are presented as mean ± SEM (**P < 0.01; n = 3). SEM, standard error of the mean.

Figure S4

Chemically modified miR-143

S: 3'-GGUCUCUACGUCGUGACGUGGAGU-5'

AS: 5'-U^G^AGAUGAAGCACUGUAGCUCA^dT^dT-3'

N: 2'-Fluoro RNA, N: 2'-O-Methyl RNA, N: RNA without modification,
^: Phosphorothioate, dT: deoxythymidine, N: mismatch

Figure S4. Scheme of CM-miR-143 development.

Red and blue letters indicate modifications with fluorine and methoxy groups in the 2′ position of the sugar ring, respectively. The black letter indicated no modification, "^" indicates phosphorothioate, and dT represents deoxythymidine. S:Sense RNA, AS:Anti-sense RNA

Table S1. Validation of the miR143 and *MARCKS* interaction

miRNA gene name (ID)	miR143 (406935)
Target gene name (ID)	*MARCKS* (4082)[a]
Species name (ID)	*Homo sapiens* (9606)
Sequence of the target region 5′-3′ (searched against TargetScan 8.0)	CAUCUC
Genomic location of MTI (microRNA-target interaction)	113860770-113860777
Genomic location of 3′ UTR[b]	191-197

[a]*MARCKS*: myristoylated alanine-rich C kinase substrate

[b]3′UTR: 3′ untranslated region
