# Supplementary material for: Antitumor effects of chemically modified miR-143 lipoplexes in a mouse model of pelvic colorectal cancer via myristoylated alanine-rich C kinase substrate downregulation
Source: Mol Ther Nucleic Acids. 2023 Nov 15;34:102079. doi: 10.1016/j.omtn.2023.102079 (PMC10783569; doi:10.1016/j.omtn.2023.102079)
Supplement: Document S2. Article plus supplemental information [file mmc2.pdf]

Molecular Therapy
Nucleic Acids
Original Article

Antitumor effects of chemically modified miR-143 lipoplexes in a mouse model of pelvic colorectal cancer via myristoylated alanine-rich C kinase substrate downregulation

Jun Arima,[1] Kohei Taniguchi,[2] Nobuhiko Sugito,[3] Kazuki Heishima,[3,4] Yoshihisa Tokumaru,[5] Yosuke Inomata,[1] Kazumasa Komura,[2] Tomohito Tanaka,[2] Masa-Aki Shibata,[6] Sang-Woong Lee,[1] and Yukihiro Akao[3]

[1]Department of General and Gastroenterological Surgery, Osaka Medical and Pharmaceutical University, 2-7, Daigaku-machi, Takatsuki, Osaka 569-8686, Japan; [2]Center for Medical Research & Development, Division of Translational Research, Osaka Medical and Pharmaceutical University, 2-7 Daigaku-machi, Takatsuki, Osaka 569-8686, Japan; [3]United Graduate School of Drug Discovery and Medical Information Sciences, Gifu University, 1-1 Yanagido, Gifu 501-1193, Japan; [4]Institute for Advanced Study, Gifu University, 1-1 Yanagido, Gifu 501-1193, Japan; [5]Department of Surgical Oncology, Graduate School of Medicine, Gifu University, 1-1 Yanagido, Gifu 501-1193, Japan; [6]Department of Anatomy and Cell Biology, Division of Life Sciences, Osaka Medical and Pharmaceutical University, 2-7 Daigaku-machi, Takatsuki, Osaka 569-8686, Japan

Replenishing tumor-suppressor miRNAs (TS-miRNAs) is a potential next-generation nucleic acid–based therapeutic approach. Establishing an effective miRNA delivery system is essential to successful TS-miRNA therapy. To overcome vulnerability to RNA nucleases, we previously developed a chemically modified miRNA143-3p (CM-miR-143). In clinical practice, colorectal cancer (CRC) pelvic recurrence is an occasional challenge following curative resection, requiring a novel therapy because reoperative surgery poses a significant burden to the patient. Hence, we considered the use of CM-miR-143 as an alternative treatment. In this study, we used a mouse model bearing pelvic CRC adjacent to the rectum and investigated the anticancer effects of CM-miR-143 lipoplexes formulated from miRNA and a cationic liposome. Compared with commercial synthetic miR-143, CM-miR-143 lipoplexes accumulated heavily in regions of the pelvic CRC tumor where the blood flow was high. As a result, systemic administration of CM-miR-143 lipoplexes improved animal survival by significantly suppressing pelvic CRC tumors and relieving a lethal bowel obstruction caused by rectal compression. Detailed protein analysis revealed that the myristoylated alanine-rich C kinase is a novel target for CM-miR-143 lipoplexes. Our results suggest that CM-miR-143 is a potential next-generation drug candidate in the treatment of CRC pelvic recurrence.

INTRODUCTION

MicroRNAs (miRNAs) are short RNA molecules comprising 19–25 nt that regulate the posttranscriptional silencing of target genes. A single miRNA can target hundreds of mRNAs and influence the expression of several genes involved in a functional interacting pathway.[1] In several human cancers, miRNAs are aberrantly expressed and play key roles in cancer initiation, development, and progression.[2,3] Consequently, miRNAs are used as clinical biomarkers and targets in drug discovery. Tumor-suppressor miRNA (TS-miRNA) therapy restores normal cellular functions by replenishing downregulated miRNAs in cancer cells.[4,5] However, because miRNAs are subject to degradation by RNA nucleases, a delivery system that prevents such degradation is required before clinical application.[6]

We investigated the chemical modification of miRNA143-3p[7–9] as a strategy for overcoming RNA nuclease activity. In general, effective chemical modifications include targeting the 2' position of the sugar ring (i.e., 2'-fluoro (2'-F), 2'-*O*-methyl, 2'-*O*-methoxyethyl, and locked nucleic acid modifications) or phosphorothioate.[6,10] We implemented these modifications to develop chemically modified miRNA143-3p (CM-miR-143), which is more resistant to RNA nuclease than the original miR-143.[2,11] Another strategy is to develop and use suitable viral or nonviral vectors for targeted miRNA delivery.[12] Lipid-based drug carriers are nonviral vectors that can penetrate the cell membrane and deliver small interfering (siRNA), mRNA, or small molecules to the target cell.[13,14] For miRNA delivery, lipoplexes are created by binding negatively charged miRNAs to positively charged lipids. Consequently, lipoplexes have been used in several applications to deliver miRNAs.[13]

Colorectal cancer (CRC) is the third most common cancer worldwide, with an incidence of 1.4 million.[15] Surgical resection is the primary treatment for CRC. Although total mesorectal excision and neoadjuvant therapy have improved CRC prognosis, pelvic recurrence occurs in 5%–13% of patients after curative resection.[16–18] Additional

Received 11 July 2023; accepted 13 November 2023;
https://doi.org/10.1016/j.omtn.2023.102079.

Correspondence: Kohei Taniguchi, Center for Medical Research & Development, Division of Translational Research, Osaka Medical and Pharmaceutical University, 2-7 Daigaku-machi, Takatsuki, Osaka 569-8686, Japan.
E-mail: kohei.taniguchi@ompu.ac.jp

This is an open access article under the CC BY license (http://creativecommons.org/licenses/by/4.0/).

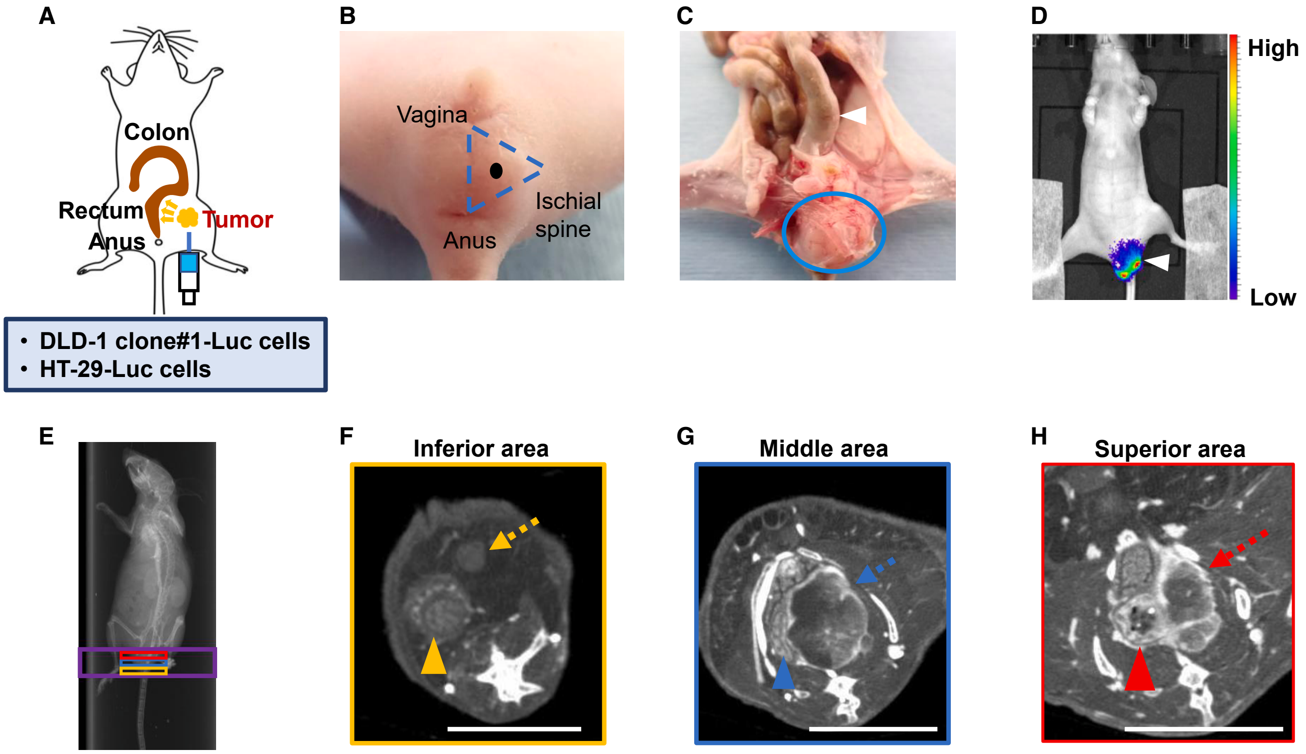

Figure 1. Features of CRC pelvic recurrence mouse model

(A) Schema of our mouse model. The needle was navigated toward the pelvic cavity ~1.0 cm from the skin, and CRC cells (DLD-1 clone#1-Luc and HT-29-Luc) were injected. The tumor grew gradually in the pelvic cavity, compressing the rectum, as the yellow arrows indicate. (B) The black point represents the injection site in the centroid of the triangle defined by the vagina, anus, and ischial spine. (C) Representative anatomical image of the mouse model. The pelvic CRC tumor is circled in blue. Pressure from the tumor caused the distension of the rectum (white arrowhead). (D) Representative IVIS images show an overall view of the mouse. The white arrowhead indicates the pelvic CRC tumor. The scale bar on the right indicates the strength of fluorescence. (E) Representative scout view. CT imaging was conducted within the area indicated by the purple square of the scout view. The yellow, blue, and red squares indicated the inferior, middle, and superior regions, respectively, of the pelvis. (F) Representative CT of the orange pelvic region. The tumor is marked by the orange arrow, and the orange arrowhead marks the rectum. The tumor did not press on the rectum in this area (scale bar: 5 mm) (G) Representative CT image of the blue pelvic region (scale bar: 5 mm). The connective tissue surrounding the CRC tumor appeared white in the CT imaging, indicating that the connective tissue was rich in blood flow (blue arrow). The rectum was deformed due to compression by the tumor (blue arrowhead). (H) Representative CT image of the red pelvic region (scale bar: 5 mm). The red arrow marks the tumor with rich blood flow in connective tissue. The red arrowhead indicates that the rectum was distended, with notable stool retention within the rectal lumen.

surgical resection is performed to control pelvic recurrence when feasible[19]; however, the procedure poses a high burden for patients because other organs (e.g., bladder, prostate, uterus, sacral bone) may also require resection. Therefore, an alternative therapeutic strategy for CRC pelvic recurrence is essential. We previously demonstrated that miR-143 is downregulated in CRC, and its restoration can effectively suppress tumors.[9] Subsequently, we focused on implementing CM-miR-143 as an alternative strategy for treating CRC pelvic recurrence.

Based on the findings of previous studies, we hypothesized that CM-miR-143 lipofection with cationic liposomes (CM-miR-143 lipoplexes) would be highly effective in treating CRC pelvic recurrence. In this study, we systemically administered CM-miR-143 lipoplexes to a CRC pelvic recurrence mouse model.[20,21] Our findings can inform the development of a novel treatment for unresectable pelvic recurrence in CRC.

RESULTS

Development of a CRC pelvic recurrence mouse model

DLD-1 clone#1-Luc or HT-29-Luc cells were concentrated to 1.0×10^6/100 μL in PBS and injected into the retroperitoneal pelvic cavity of each mouse (Figure 1A). As previously reported, the injection site was selected within the triangle defined by the vagina, anus, and ischial spine (Figure 1B).[20,21] We sacrificed mice 2 weeks after CRC engraftment and examined the pelvic anatomy. Pelvic CRC occupied the pelvic cavity, and the rectum was distended (Figure 1C). The location and volume of the pelvic CRC tumor were evaluated by an *in vivo* imaging system (IVIS), revealing that the pelvic CRC tumor was localized in the pelvis without metastasizing to other organs (Figure 1D). Moreover, computed tomography (CT) imaging revealed that the pelvic CRC tumor compressed the rectum in the pelvis, and the blood flow was high in the connective tissue surrounding the tumor (Figures 1E–1H). These findings suggest that the pelvic CRC tumor occupied the pelvic cavity, caused a life-threatening

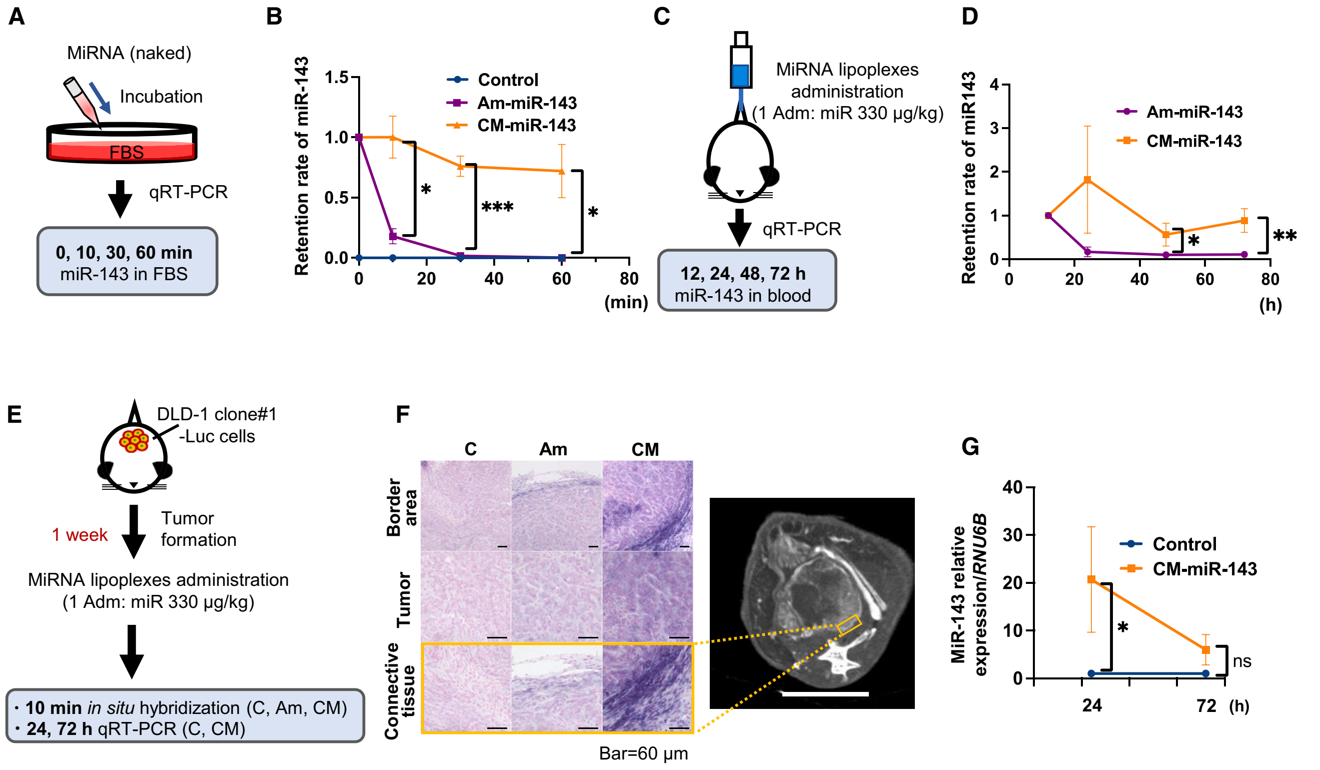

Figure 2. Stability of CM-miR-143 lipoplexes in mouse blood and delivery to pelvic CRC tumors

(A) Scheme of *in vitro* experiments for measuring miR-143 levels using qRT-PCR. Naked miRNA was incubated in FBS. Retention rates of miR-143 were measured at 0, 10, 30, and 60 min after incubation. (B) Blue, purple, and orange lines indicate control (C) miRNA, Am-miR-143 (Am), and CM-miR-143 (CM), respectively. Values at 0 min for Am-miR-143 and CM-miR-143 are indicated as 1.0. After incubation for 10 min, Am-miR-143 levels decreased significantly. CM-miR-143 levels remained high until 60 min after incubation. Data are presented as mean ± SEM (*p < 0.05; ***p < 0.001; n = 3). (C) Scheme of *in vivo* experiments for measuring plasma miR-143 levels using qRT-PCR. Each miRNA lipoplex (330 μg/kg miRNA per administration) was injected into the tail vein once. Blood miR-143 levels were measured at 12, 24, 48, and 72 h after administration. (D) Purple and orange lines indicate miR-143 ratios in the Am-miR-143 and CM-miR-143 groups, respectively. miR-143 levels at 12 h are indicated as 1.0. At 24 h after administration, Am-miR-143 levels decreased, whereas CM-miR-143 levels remained high until 72 h. Data are presented as the mean ± SEM (*p < 0.05; **p < 0.01; n = 7). (E) Scheme of *in vivo* experiments for *in situ* hybridization and qRT-PCR, aiming to characterize miR-143-143 distribution in pelvic CRC tumors (DLD-1 clone#1-Luc cells). *In situ* hybridization was performed using tissue samples from pelvic CRC tumors resected 10 min after miRNA lipoplex administration; qRT-PCR was performed 24 and 72 h after administration (330 μg/kg miRNA per administration). (F) Representative images from *in situ* hybridization and CT. Purple signals *in situ* hybridization indicate miR-143. The images illustrate 3 regions: the border area, tumor, and connective tissue *in situ* hybridization. The border area represents the region surrounding the tumor. The connective tissue, highlighted by an orange square in CT imaging, is a magnified image of the border area. CT imaging showed that blood flow was rich in connective tissue (scale bar: 5 mm). (G) Results from qRT-PCR revealed that miR-143 levels were significantly higher at 24 h in the CM-miR-143 group than in the control group. However, the 2 groups did not differ significantly in miR-143 levels by 72 h. Data are presented as mean ± SEM (*p < 0.05; n = 4). ns, not significant; Adm, administration.

bowel obstruction by compressing the rectum, and was nourished by the surrounding blood flow.

In vitro CM-miR-143 stability and *in vivo* tissue distribution of CM-miR-143 lipoplexes

In fetal bovine serum (FBS), naked CM-miR-143 had a significantly higher retention rate than naked Am-miR-143 after incubation for 10, 30, and 60 min (Figures 2A and 2B). When miR-143 lipoplexes were systemically administered to mice using Invivofectamine 3.0 (Life Technologies, Carlsbad, CA), the retention rates at 48 and 72 h were significantly higher in the CM-miR-143 group than in the Am-miR-143 group (Figures 2C and 2D). These findings indicate that CM-miR-143 is more resistant to RNA nucleases than Am-miR-143 and, therefore, remains longer in blood.

Next, we used *in situ* hybridization and qRT-PCR to assess miR-143 distribution in pelvic CRC tumors after administering CM-miR-143 lipoplexes (Figure 2E). The results showed that the distribution in pelvic CRC tumors and the surrounding connective tissue was higher following CM-miR-143 administration than Am-miR-143 administration. Moreover, CM-miR-143 accumulation was higher in the connective tissue, where CT imaging indicated higher vascularity compared with the inner tumor portions (Figure 2F). At 24 h postadministration, miR-143 levels were significantly higher in the CM-miR-143 group than in the control group, although this difference gradually decreased by 72 h (Figure 2G). Hence, CM-miR-143 lipoplexes can deliver more miR-143 to pelvic CRC tumors through the bloodstream than Am-miR-143 lipoplexes, and multiple sequential administrations are required to maintain the basal therapeutic level of miR-143.

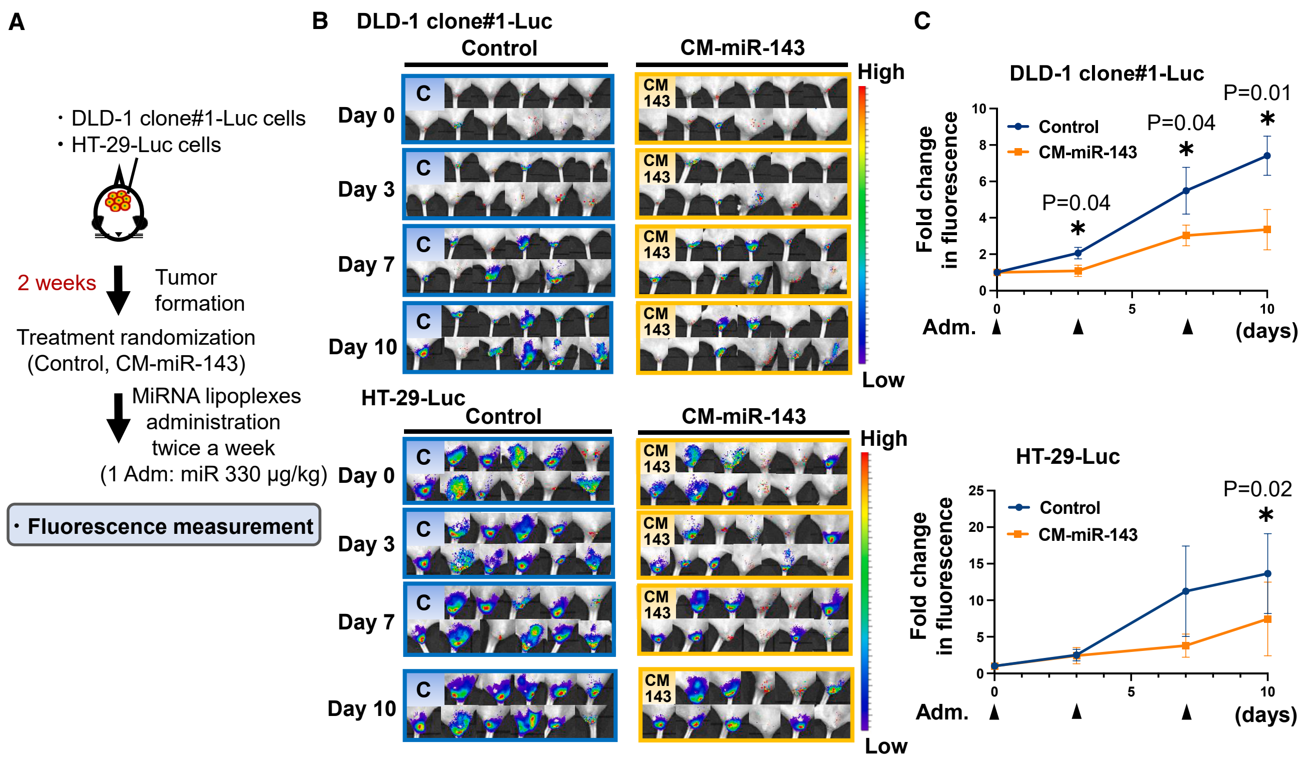

Figure 3. Systemic administration of CM-miR-143 lipoplexes suppressed tumor growth of CRC pelvic recurrence mouse

(A) Scheme of *in vivo* experiments to measure fluorescence. Mice were randomly divided into 2 groups after tumor formation. To measure fluorescence, miRNA lipoplexes (330 μg/kg miRNA per administration) were injected into the tail vein 3 times over 10 days. (B) All IVIS images of control (C) and CM-miR-143 (CM-143) at days 0, 3, 7, and 10 posttreatment in the DLD-1 clone#1-Luc mouse model and the HT-29-Luc mouse model are shown. (C) Fluorescence intensity was measured on days 0, 3, 7, and 10 posttreatment using IVIS in DLD-1 clone#1-Luc and HT-29-Luc mouse models (DLD-1 clone#1-Luc: n = 11, HT-29-Luc: n = 11). The graph shows variation in fluorescence intensity over time. Fold changes in fluorescence intensity were analyzed per group. In the DLD-1 clone#1-Luc mouse model, the CM-miR-143 group experienced significantly suppressed fluorescence intensity on days 3, 7, and 10 posttreatment compared with the control group; in the HT-29-Luc mouse model, significant suppression occurred only on day 10. Black arrowheads indicate the injection of miRNA lipoplexes into the tail vein. Data are presented as mean ± SEM (*$p < 0.05$).

CM-miR-143 prolonged the survival of CRC pelvic recurrence mice by suppressing tumor growth

We examined the antitumor effects of CM-miR-143 on two CRC pelvic recurrence mouse models (Figure 3A) established by implanting either DLD-1 or HT-29 cells containing the luciferase gene. Tumor volume was estimated by measuring luciferase activity. Results from IVIS showed that CM-miR-143 lipoplexes significantly reduced fluorescence intensity in DLD-1 clone#1-Luc and HT-29-Luc pelvic CRC mouse models (Figures 3B and 3C). To further evaluate the antitumor effect, we assessed the survival of CRC pelvic recurrence mice. No significant difference was observed in the fluorescence or body weight between groups compared with those before treatment initiation (Figures 4A–4C). Kaplan-Meier plots showed that the CM-miR-143 group survived longer than the control group for both mouse models (Figure 4D). The body weights of the mice did not differ significantly between the control and CM-miR-143 groups after treatment (Figure 4E). Pathological changes in the liver and kidney were not observed in the untreated (not administered miRNA or Invivofectamine 3.0), control (administered control miRNA), or CM-miR-143 groups (Figure S1). These findings suggest that CM-miR-143 lipoplexes suppress pelvic CRC tumor growth, relieving tumor-induced compression on the rectum and improving survival without severe side effects.

CM-miR-143 induced antitumor effects by downregulating myristoylated alanine-rich C kinase substrate *in vivo*

Next, we investigated the mechanism underlying the anticancer effects of CM-miR-143. To this end, we performed proteome and immunohistochemical (IHC) analyses on tissue samples from the pelvic CRC tumors of DLD-1 clone#1-Luc mice. In addition, western blotting was performed on tumor samples from both mouse models (DLD-1 clone#1-Luc and HT-29-Luc) after treatment with CM-miR-143 lipoplexes (Figure 5A). Several proteins were significantly downregulated in the CM-miR-143 group (Figures 5B and 5C), and of these, only myristoylated alanine-rich C kinase substrate (MARCKS) contained a binding site for miR-143 (Table 1). IHC analysis confirmed that MARCKS was downregulated in pelvic CRC tumors (Figure 5D). In addition, western blot analysis revealed phospho (p)-MARCKS and MARCKS downregulation in both cell types (Figures 5E and 5F). The phosphatidylinositol 3-kinase/AKT

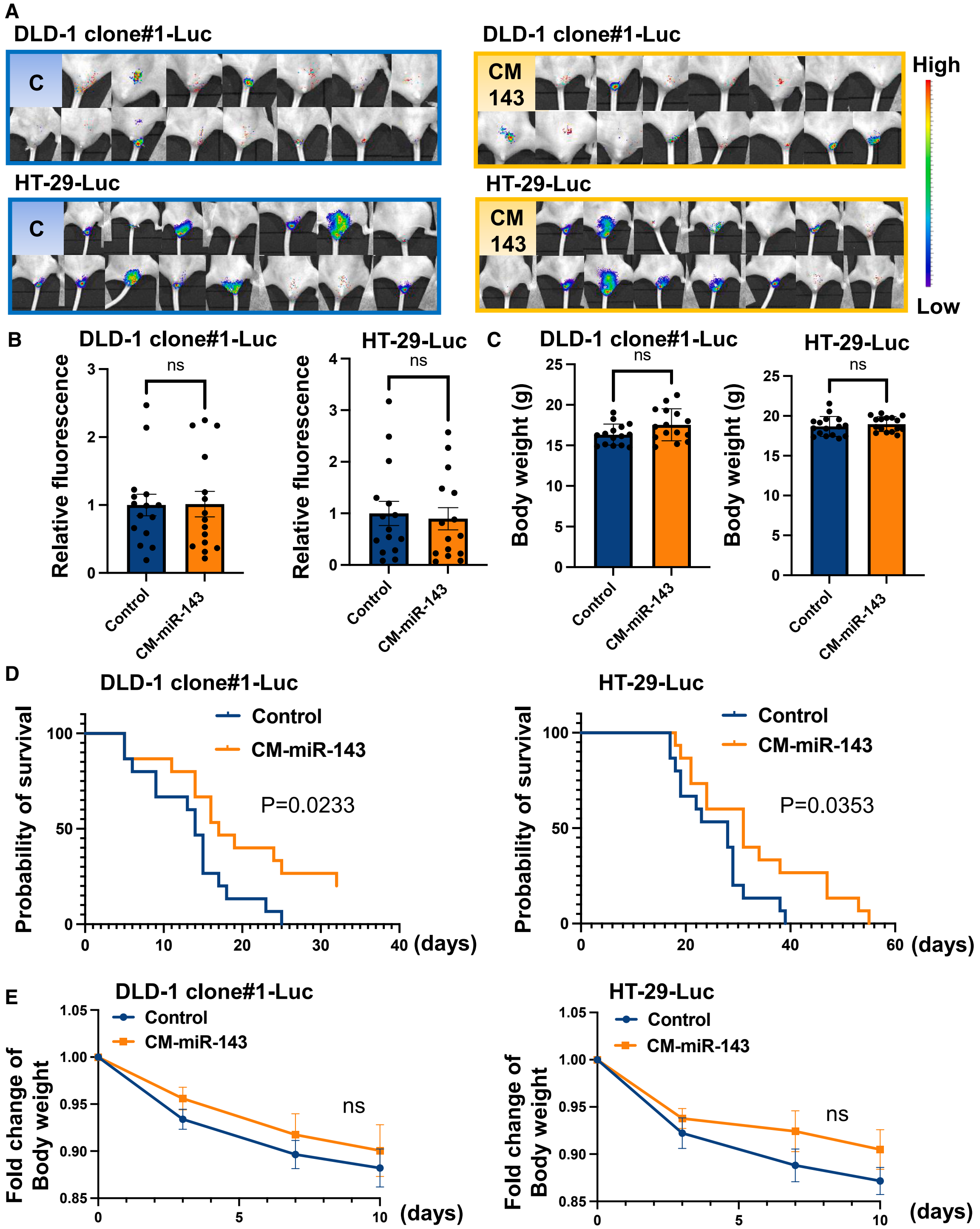

Figure 4. Systemic administration of CM-miR-143 lipoplexes prolonged CRC pelvic recurrence mouse survival

(A) All IVIS images of control and CM-miR-143 before treatment in the DLD-1 clone#1-Luc and HT-29-Luc mouse models. (B) The relative fluorescence intensity of control (C) and CM-miR-143 (CM-143) before treatment in the DLD-1 clone#1-Luc and HT-29-Luc mouse models. There was no significant difference between the 2 groups. (n = 15)

(legend continued on next page)

pathway, implicated in cancer growth signaling, is related to p-MARCKS and MARCKS.[22] Hence, we also examined p-AKT and AKT expression to determine their relationship with MARCKS; however, we observed no significant differences (Figures 5E and S2). These findings suggest that MARCKS downregulation may contribute to CM-miR-143-induced pelvic CRC tumor suppression.

CM-miR-143 targeted MARCKS to inhibit CRC cell proliferation *in vitro*

TargetScan 8.0 (https://www.targetscan.org/vert_80/) and GeneCards (https://www.genecards.org) showed that the 3' UTR of *MARCKS* contains binding sites for miR-143 (Table S1). Hence, we prepared *MARCKS* 3' UTR wild-type (WT) and mutated type (MUT) plasmids (Figure 6A). The luciferase reporter activity of WT *MARCKS* was significantly inhibited after treatment with Am-miR-143; however, mutating the *MARCKS* 3′ UTR-binding site abolished this inhibitory activity (Figure 6B). Moreover, qRT-PCR indicated that *MARCKS* mRNA expression was downregulated 48 h after the transfection of DLD-1 and HT-29 cells with CM-miR-143 *in vitro* (Figure S3). CM-miR-143 significantly inhibited CRC cell proliferation in both cell types compared to control miRNA and Am-miR-143 (Figure 6C). Western blotting confirmed that CM-miR-143 suppressed p-MARCKS and MARCKS abundance. In contrast, p-AKT and AKT expression levels were downregulated *in vitro* but not *in vivo* (Figure 6D). In addition, siRNA-MARCKS 1 and 2 inhibited CRC cell proliferation in both cell types compared to the control (Figure 6E). Western blotting revealed that siRNA-MARCKS 1 and 2 downregulated the abundance of MARCKS but not that of AKT (Figure 6F). These findings suggest that CM-miR-143 inhibits CRC cell proliferation by suppressing MARCKS and AKT, although knocking down MARCKS did not directly suppress AKT expression.

DISCUSSION

In this study, we systemically administered CM-miR-143 lipoplexes to a CRC pelvic recurrence mouse model and successfully evaluated the therapeutic potential of CM-miR-143 as an anticancer agent. The results showed that the CM-miR-143 lipoplexes significantly improved the survival of the mouse model by suppressing pelvic CRC tumor growth; the underlying mechanism partially involves MARCKS downregulation.

We selected commercial Invivofectamine 3.0 as a drug delivery system and generated CM-miR-143 lipoplexes to counteract the effects of RNA nucleases. Invivofectamine 3.0 is a next-generation lipid-based carrier for *in vivo* research and has been used to deliver siRNA or miRNA to the liver and subcutaneous tumors.[23,24] Here, Invivofectamine 3.0 was not an appropriate carrier for the systemic administration of commercial synthetic miR-143 (Am-miR-143) to our pelvic CRC mouse model. Based on the qRT-PCR analysis of blood samples, the Am-miR-143 lipoplexes, without chemical modifications, exhibited instability in the mouse bloodstream, potentially due to its rapid degradation. Furthermore, *in situ* hybridization results revealed that Am-miR-143 lipoplexes were not effectively delivered to the pelvic CRC tumors postadministration, indicating a limitation in their uptake within the tumor tissues. However, we observed that CM-miR-143 lipoplexes with Invivofectamine 3.0 were stable in mouse blood and sufficiently delivered miR-143 to pelvic CRC tumors through the bloodstream. Thus, lipoplexes formed between CM-miR-143 and Invivofectamine 3.0 cationic liposomes could be used as drug-delivery systems that target tumor tissues.

The CRC pelvic recurrence mouse model is commonly used to verify the effectiveness of novel treatments, including TS-miRNA therapy.[20,21,25] Although a subcutaneous CRC mouse model is easier to establish and monitor, it cannot replicate the original anatomic site of CRC.[26] Indeed, subcutaneous CRC switches phenotypes and frequently fails to progress and metastasize due to microenvironmental differences.[27,28] Furthermore, tumor responses to therapy can vary considerably depending on whether the cancer cells are implanted in an ectopic (subcutaneous) or orthotropic location.[29] Accordingly, we selected the CRC pelvic recurrence mouse model for our analyses.

Our previous studies showed that CM-miR-143 suppresses the proliferation of CRC, gastric, and bladder cancers by specifically repressing the rat sarcoma virus (RAS) network.[11,30] Downregulation of the RAS network inhibits KRAS, son of sevenless homolog 1, AKT, and extracellular signal-regulated kinase.[8,31] To establish the expression profile of all of the proteins dysregulated by CM-miR-143 treatment, we analyzed the proteomes of tumor samples after the systemic administration of CM-miR-143 lipoplexes. CM-miR-143 downregulated MARCKS expression *in vivo,* which was confirmed *in vitro*. These results imply that *MARCKS* is the target gene of CM-miR-143. To support our hypothesis, analysis with TargetScan 8.0 revealed that among the downregulated proteins identified in our proteome analysis, only MARCKS has a direct binding site for miR-143 (Table 1).

Identified as a major target of protein kinase C, MARCKS is a membrane-associated protein[32] that is an attractive target for cancer therapy,[22] given its association with the growth, proliferation, motility, and invasion of multiple cancers, including lung cancer and glioblastoma.[33,34] In fact, previous reports have shown that TS-miRNAs, such as miR-34c-3p and miR-188, exert their anticancer effects by silencing *MARCKS* in the liver and prostate.[35,36] These reports agree with our results, showing that CM-miR-143 inhibits CRC proliferation via *MARCKS* knockdown. Moreover, the MARCKS family member MARCKS-like protein 1 (MARCKSL1) was downregulated (Table 1). As homologs, MARCKS and MARCKSL1 have highly

(C) The body weight of control and CM-miR-143 before treatment of the DLD-1 clone#1-Luc and HT-29-Luc mouse models. There was no significant difference in body weight between the 2 groups (n = 15). (D) Kaplan-Meier plots show the survival rate in control (blue line) and CM-miR-143 (orange line) groups in the DLD-1 clone#1-Luc and HT-29-Luc mouse models (n = 15). The significance of survival duration was determined using log rank survival analysis. (E) Body weights did not differ significantly between control and CM-miR-143 groups in either the DLD-1 clone#1-Luc or the HT-29-Luc mouse models after treatment (n = 15). ns, not significant. Data are presented as the mean ± SEM.

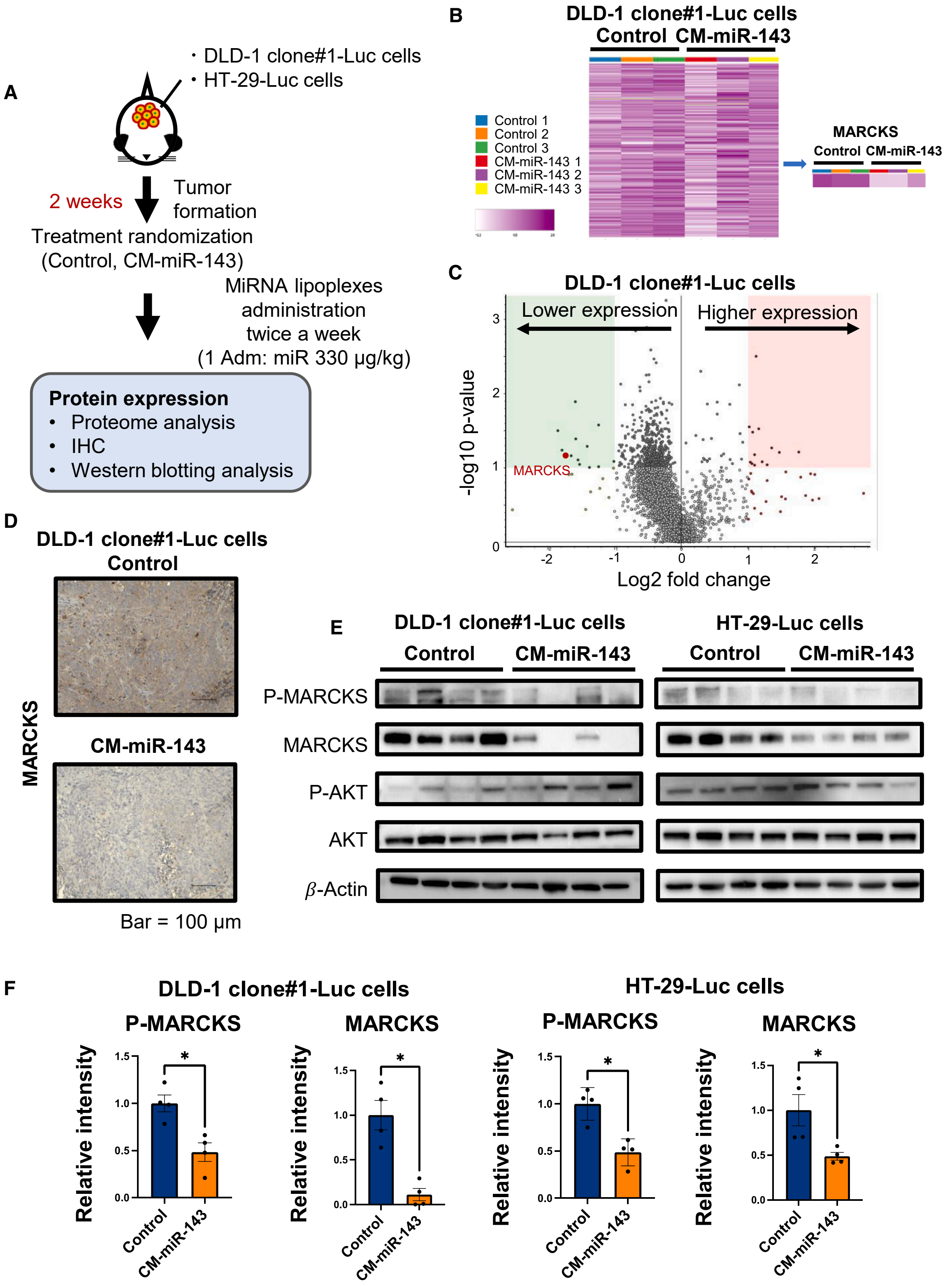

(legend on next page)

conserved regions and similar functions related to the regulation of cell migration.[37] Hence, the observation that MARCKSL1 was also downregulated confirms the accuracy of our protein analysis results.

MARCKS phosphorylation activates the AKT-mediated signaling pathway, leading to cell growth and proliferation.[22] P-MARCKS knockdown also inhibits lung cancer progression by downregulating AKT.[38] However, we did not find p-MARCKS or AKT to be significantly associated with CRC. This outcome suggests that the inhibition of CRC proliferation via p-MARCKS suppression was independent of AKT. Therefore, the function of MARCKS and its relationship with other cancer-related genes warrants further investigation.

This study has certain limitations. First, we did not investigate immune-related effects in this mouse model. However, severe adverse immune-related effects were reported during the Phase I clinical trial of miR-34a.[39] Such effects should be studied in other animal models to determine how they can be mitigated. Second, we only evaluated the pathological effects on the liver and kidneys; hence, other organs should also be assessed in future studies. Moreover, liver and kidney functions should be evaluated further using biochemical blood tests.

In conclusion, systemic administration of CM-miR-143 lipoplexes improved mouse survival by significantly suppressing pelvic CRC growth. Moreover, MARCKS represented a major target gene of CM-miR-143 *in vivo* and *in vitro*. Collectively, our findings can help overcome CRC pelvic recurrence and encourage clinical implementation of TS-miRNA replacement therapy.

MATERIAL AND METHODS

CRC cell lines

Human CRC cell lines stably expressing luciferase (DLD-1 clone #1-Luc: JCRB1382, RRID: CVCL J250 and HT-29-Luc: JCRB1383, RRID:CVCL J256) were obtained from the Japanese Cancer Research Resources Bank (Suita City, Osaka, Japan). Human CRC cell lines (DLD-1, RRID: CVCL 0248 and HT-29, RRID: CVCL 0320) were obtained from Taiho Pharmaceutical (Shibuya-ku, Tokyo, Japan). DLD-1 clone #1-Luc, DLD-1, and HT-29 cells were cultured in RPMI 1640 and Phenol Red (Fujifilm Wako, Chuo-ku, Osaka, Japan), and HT-29-Luc cells were cultured in McCoy's 5A medium. All of the media were supplemented with 10% (v/v) heat-inactivated FBS (Hyclone Laboratories, Logan, UT) and incubated at 37°C and 5% CO_2.

Preparation of miRNAs

Three miRNA types were used: control miRNA, mature miR-143 (Am-miR-143, a commercially available synthetic miR-143) (mirVana miRNA mimic; Ambion, Foster City, CA), and CM-miR-143. The control was a nonspecific miRNA (Hokkaido System Science, Sapporo, Hokkaido, Japan) with a 5'-GUA GGA GUA GUG AAA GGC C-3' sequence.[11] CM-miR-143 was chemically modified with fluorine and a methoxy group in the 2' position of the sugar ring, phosphorothioate, and deoxythymidine in the passenger strand (Figure S4). miRNA lipoplexes (control, Am-miR-143, or CM-miR-143) were prepared using Invivofectamine 3.0 following the manufacturer's protocol.

Mouse model of CRC pelvic recurrence

Seven-week-old female BALB/c nude mice were acquired from Japan SLC (Hamamatsu-shi, Shizuoka, Japan). Mice were housed in plastic cages with woodchip bedding and maintained under controlled temperature (21°C ± 2°C), humidity (50% ± 10%), and lighting (12 h/12 h light/dark cycle). No more than five mice were housed in one cage. Food and water were provided *ad libitum*. All of the mice were acclimatized for 1 week before test initiation. All of the animal procedures were performed in accordance with the Guide for Care and Use of Laboratory Animals of the National Institutes of Health. The protocol was approved by the Osaka Medical and Pharmaceutical University Animal Care and Use Committee (approval no. 21024-A).

IVIS

Each mouse was injected intraperitoneally with 3 mg of D-luciferin potassium salt (Wako Pure Chemical Corporation, Kita-ku, Osaka, Japan). Fluorescence signals in the pelvic CRC mouse model were quantified using IVIS and Living Image Software version 4.0 (PerkinElmer, Waltham, MA). Tumor fluorescence intensity was quantified using the region of interest control tool.

CT

Pelvic CRC was detected using a Latheta CT scanner (LCT-200 series, Hitachi, Tokyo, Japan) with a contrast agent. This CT system

Figure 5. CM-miR-143 lipoplexes downregulated MARCKS expression *in vivo*

(A) Scheme of *in vivo* experiment for proteome, IHC, and western blot analyses. In the DLD-1 clone#1-Luc model, miRNA lipoplexes (330 μg/kg miRNA per administration) were injected into the tail vein 4 times in 2 weeks before tissue samples were obtained from pelvic CRC tumors for the 3 analyses. In the HT-29-Luc model, miRNA lipoplexes (330 μg/kg miRNA) were administered twice in 1 week before tissue samples from pelvic CRC tumors were obtained for western blotting. (B) Heatmap showing results of 4,158 proteins from proteome analysis of DLD-1 clone#1-Luc tumor samples. Heatmap showing the results of 3 samples in the control and CM-miR-143 groups. The purple intensity indicates the expression of the protein. The right-side panel indicates that the expression of MARCKS is downregulated. (C) Volcano plot showing results from proteome analysis of control and CM-miR143 groups (n = 3). The red point indicates MARCKS expression. Negative log2 fold change: higher expression in the control group and lower expression in the CM-miR143 group. Positive log2 fold change: lower expression in the control group and higher expression in the CM-miR143 group. (D) Representative IHC images for MARCKS in control and CM-miR-143 groups. MARCKS downregulation was observed in the CM-miR-143 group. (E) Western blotting to evaluate p-MARCKS, MARCKS, p-AKT, and AKT expression. p-MARCKS and MARCKS protein expression was significantly downregulated in the CM-miR-143 group. p-AKT and AKT levels did not differ between the control and CM-miR-143 groups. β-Actin was used as an internal control. (F) p-MARCKS and MARCKS band intensities in the western blots were calculated using ImageJ. MARCKS and p-MARCKS expression was significantly downregulated in pelvic CRC tumors in the DLD-1 clone#1-Luc and HT-29-Luc models. Data are presented as mean ± SEM (*$p < 0.05$; n = 4).

Table 1. List of downregulated proteins according to proteome analysis

Gene symbol	Gene name	Prognostic markers in cancer[a]	Protein function[a]	Binding sites of miR-143[b]	Abundance ratio: CM-143/C[c]
PTMA	Prothymosin alpha	Renal cancer, liver cancer, and cervical cancer	Mediate immune function	0	0.277
PTMS	Parathymosin	Renal cancer	Immunity	0	0.29
**MARCKS**	**Myristoylated alanine-rich C kinase substrate**	**Liver cancer and renal cancer**	**Actin binding, calmodulin binding**	**1**	**0.299**
IGKV4-1	Immunoglobulin kappa variable 4-1	Renal cancer, breast cancer, and head and neck cancer	Adaptive immunity, immunity	0	0.311
MARCKSL1	MARCKS-related protein	Liver cancer and renal cancer	Actin binding, calmodulin binding	0	0.319
PSAP	Prosaposin	None	Lipid metabolism, sphingolipid metabolism	0	0.322
PCNP	PEST proteolytic signal-containing nuclear protein	Liver cancer	Cell cycle	0	0.333
HIST1H1C	Histone H1.2	Urothelial cancer, pancreatic cancer, and renal cancer	DNA binding	0	0.334
CD63	CD63 antigen	Liver cancer	Protein transport, transport	0	0.343
SMG8	Protein SMG8	Liver cancer	Nonsense-mediated mRNA decay	0	0.348

[a]Prognostic markers for cancer and protein functions were determined using The Human Protein Atlas (https://www.proteinatlas.org/ENSG00000187514-PTMA).
[b]Binding sites of miR-143 were found using TargetScan 8.0.
[c]C, control group; CM-143, CM-miR-143 group.

provides a tube power voltage of 50 kV and a tube current of 0.5 mA. The mean exposure time was 11 min for an average of 100 scans. The image matrix was 1024 × 1024, and the voxel size was 48 × 192 μm. Images were reconstructed using traditional filter functions.

qRT-PCR

To measure miRNA expression, total RNA was reverse transcribed to cDNA using the TaqMan MicroRNA Reverse Transcription Kit (Applied Biosystems, Foster City, CA). To assess Am-miR-143 expression, we used TaqMan MicroRNA Assays (Applied Biosystems) and the THUNDERBIRD probe qPCR Mix (Toyobo, Kita-ku, Osaka, Japan). To measure mRNA, total RNA was reverse transcribed to cDNA using the PrimeScript RT Reagent Kit (Takara, Kusatsu City, Shiga, Japan), and *MARCKS* mRNA expression levels were determined using the THUNDERBIRD SYBR qPCR Mix (Totobo, QPS-201) with *MARCKS* and *GAPDH* (glyceraldehyde 3-phosphate dehydrogenase) primers. All of the experiments using kits were performed following the manufacturer's protocol. The Ct was defined as the fractional cycle number at which fluorescence exceeded a specified level. The internal controls in tissue and blood samples were *RNU6B* and miR-16 for miRNAs. Meanwhile, the internal control for *in vitro* mRNA analysis was *GAPDH*. The Ct value was calculated using the second derivative maximum method, and relative quantification was performed using the comparative Ct method. All of the experiments were performed in triplicate. The PCR primers for miR-143, *RNU6B*, *MARCKS*, and miR-16 were obtained from Applied Biosystems. The PCR primers for *GAPDH* were obtained from Bio-Rad (Hercules, CA).

Assessment of miRNA stability

For *in vitro* experiments, control miRNA, Am-miR-143, and CM-miR-143 (1 μL of 10 μM) were incubated in 100 μL of FBS (Hyclone Laboratories) at 37°C and 5% CO_2 for 0, 10, 30, and 60 min. Next, miRNA was isolated and quantified using qRT-PCR. For *in vivo* experiments, Am-miR-143 and CM-miR-143 lipoplexes were injected into the tail veins of mice using a 30G needle. For each administration, miRNA concentration was adjusted to 330 μg/kg in PBS (100 μL). Blood (10 μL) was collected from tail veins at 12, 24, 48, and 72 h after administering the miRNA-lipoplex and mixed with EDTA on ice. Blood samples were centrifuged twice at 3000 × *g* and 4°C to separate blood cells. The plasma (supernatant) was collected to assess miRNA expression using qRT-PCR.

In situ hybridization of miRNA

Mice were euthanized 10 min after administering control miRNA, Am-miR-143, or CM-miR-143 lipoplexes (330 μg/kg miRNA per administration). Pelvic CRC tumors were resected, frozen in liquid nitrogen, sliced sequentially into 10-μm sections, fixed in 4% paraformaldehyde for 2 h at room temperature, and then fixed for 20 min at 4°C. Tissues were washed 4 times with RNase-free PBS. The miR-143 probe (1 μL; YD00610001, Qiagen, Hilden, Germany) was denatured for 4 min at 90°C and mixed with a hybridization buffer (600 μL) *in situ* for 1 h at 53°C. Slides were washed at room temperature 3 times (5 min each) in 0.2% saline sodium citrate and once in Tris-buffered saline (TBS) containing 0.1% Tween 20. Slides were initially blocked for 10 min with normal horse serum and then incubated for 1 h at 37°C with an anti-DIG-AP antibody diluted in normal horse serum (1:50). Subsequently, the slides were washed 3 times in TBS

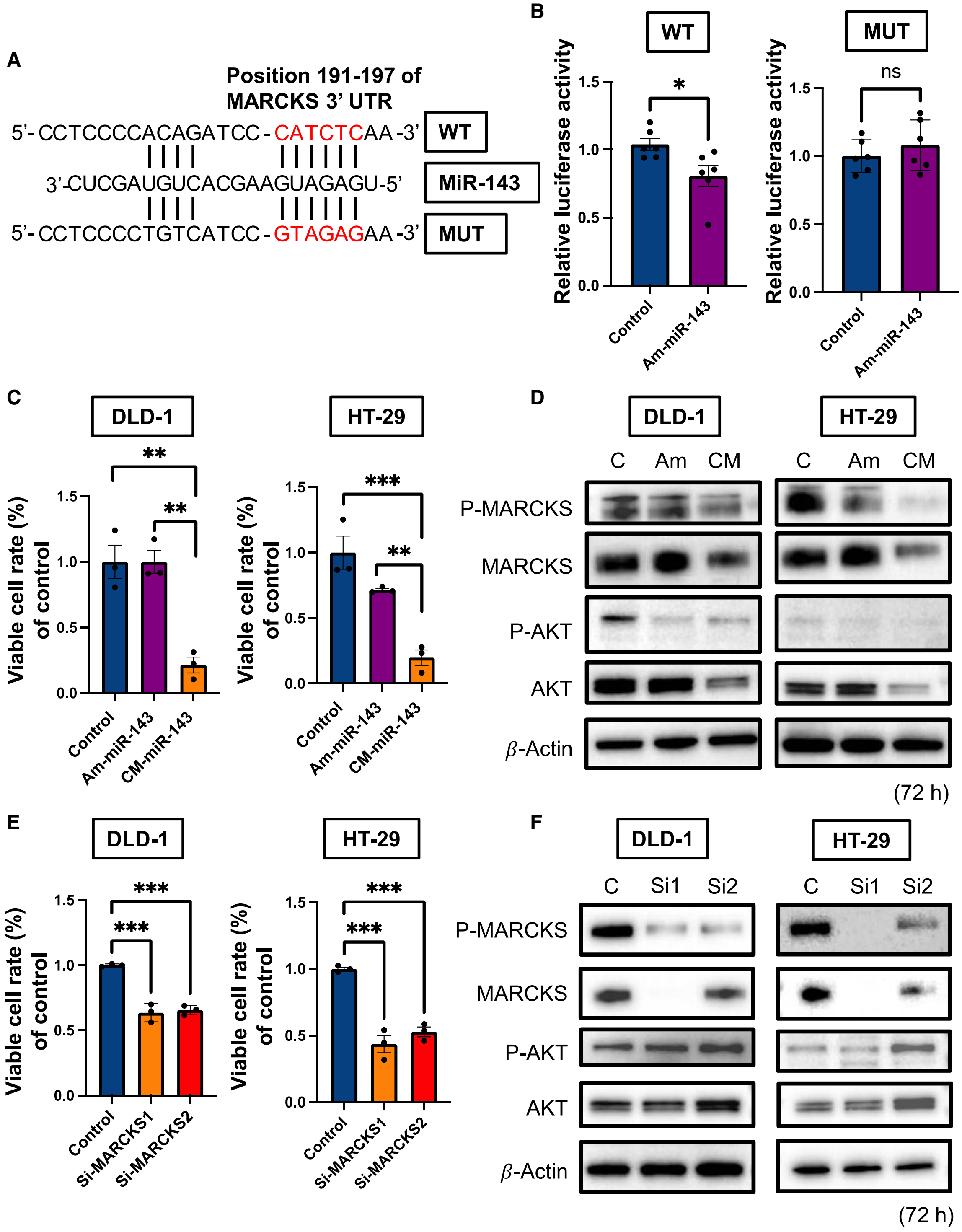

Figure 6. CM-miR-143 inhibited CRC cell proliferation by downregulating MARCKS *in vitro*

(A) Schematic of putative miR-143 seed sequence binding at the *MARCKS* 3' UTR region (WT) and MUT sequence of the putative binding site. Red letters are binding sites and mutation sequences used for luciferase assays in MUT. (B) Am-miR-143 significantly inhibited luciferase activity in WT. Activities were completely recovered to the control levels in MUT. Data are presented as mean ± SEM (*$p < 0.05$; n = 6). ns, not significant. (C) Cell viability at 72 h after transfection of each miRNA (10 nM) with lipofectamine RNAiMAX in the DLD-1 or HT-29 cells. CM-miR-143 significantly suppressed cell viability in both cell lines compared with control miRNA or Am-miR-143. Data are presented

(legend continued on next page)

containing 0.1% Tween 20. Slides were further immersed in NBT/BCIP solution (Roche Ready Mix tablets, Roche, Basel, Switzerland) with 0.2 mM levamisole for 2 h. Subsequently, slides were washed twice with KTBT buffer for 2 min and then with distilled water. Slides were immersed in Nuclear Fast Red for 1 min and passed through distilled water twice. Finally, the slides were dehydrated, permeated, and sealed. Images were captured under a BZ-x700 microscope (Keyence, Shinjuku-ku, Tokyo, Japan).

Assessment of miR-143 expression in pelvic CRC tumors

Four mice with pelvic CRC were euthanized 24 and 72 h after administering control miRNA or CM-miR-143 lipoplexes (330 μg/kg miRNA per administration). Pelvic CRC was resected, and total RNA was extracted using TRIzol containing phenol/guanidium isothiocyanate (Invitrogen, Waltham, MA). The expression of miR-143 was evaluated via qRT-PCR.

Fluorescence imaging of pelvic CRC tumors

To investigate the anticancer effect of CM-miR-143, fluorescence intensities were measured with IVIS 0, 3, 7, and 10 days after injecting the control miRNA or CM-miR-143 lipoplexes (330 μg/kg miRNA) in DLD-1 clone#1-Luc (n = 11) and HT-29-Luc (n = 11) CRC pelvic recurrence mice. The control was a nonspecific miRNA with a 5'-GUA GGA GUA GUG AAA GGC C-3' sequence.

Survival analysis of pelvic CRC mouse models

Mice were treated with control miRNA or CM-miR-143 after confirming that the two treatments caused no significant changes in the CRC volume or body weight. In the control miRNA and CM-miR-143 groups, miRNA lipoplexes (330 μg/kg miRNA) were injected into the tail vein twice weekly until the endpoint (n = 15). Body weights were measured twice weekly. The control was a nonspecific miRNA with a 5'-GUA GGA GUA GUG AAA GGC C-3' sequence.

Proteome analysis

Three samples of pelvic CRC (DLD-1 clone#1-Luc cell) in the control and CM-miR-143 groups were frozen in liquid nitrogen. After the miRNA lipoplex was administered four times, samples were resected, extracted, and refined to isolate proteins 3 days after the final administration. Isolated proteins were digested with trypsin and labeled with Tandem Mass Tag (TMT). Proteome analysis was conducted using quantitative liquid chromatography-tandem mass spectrometry (MS/MS) of TMT-labeled peptides (Integrale, Tokyo, Japan). Each peptide fraction was analyzed with Q Exactive Plus (Thermo Fisher Scientific, Waltham, MA) coupled online with a capillary high-performance liquid chromatography system (EASY-nLC 1200, Thermo Fisher Scientific) to acquire its MS/MS spectra. Heatmaps and volcano plots were generated using Proteome Discoverer (version 2.4, Thermo Fisher Scientific, RRID: SCR_014477).

Histological and IHC analyses

Specimens were fixed with 10% formaldehyde in phosphate buffer and embedded in paraffin (Nara-byouri Laboratory, Yamatokoriyama City, Nara, Japan). Sections (4 μm thick) were mounted on adhesive glass slides, deparaffinized using xylene, and hydrated with an ethanol series. For histological assessment, sections were stained with H&E (Muto Pure Chemical, Chiyoda-ku, Tokyo, Japan). For IHC, endogenous peroxidase was blocked with 10% H_2O_2 and Serum-Free Ready-to-Use Protein block (Dako, Santa Clara, CA). Tissue specimens were incubated with anti-MARCKS antibody (1:100 dilution; catalog no. 5607, Cell Signaling Technology, Santa Cruz, CA) overnight at 4°C. Next, tissue sections were washed with PBS and incubated with a secondary antibody labeled with the Envision Dual Link System-HRP (Dako) at 37°C for 30 min. Immunoreactions were visualized using 3,3'-diaminobenzidine solution (Nichirei Bioscience, Chuo-ku, Tokyo, Japan) and counterstained with hematoxylin. Finally, sections were dehydrated and mounted. Images were captured under a BZ-x700 microscope (Keyence).

Western blot analysis

Samples were obtained 3 days after administering the control miRNA or CM-miR-143 lipoplexes 4 times twice per week to DLD-1 clone#1-Luc mice and 2 times twice per week to HT-29-Luc mice. Samples from *in vitro* experiments were obtained 72 h after miRNA and siRNA transfection with lipofectamine RNAiMAX (Invitrogen), following the manufacturer's protocol. All of the samples were homogenized in RIPA Lysis and Extraction Buffer (Life Technologies) with a 2% protease inhibitor cocktail and a 2% phosphatase inhibitor cocktail 2/3 (Sigma-Aldrich, St. Louis, MO) and incubated for 20 min on ice. After centrifugation at 12,000 × g and 4°C for 20 min, supernatants were collected as whole-cell protein samples. The protein content was measured using the DC Protein Assay Kit (Bio-Rad). The proteins in the lysate (6–10 μg) were separated with SDS-PAGE using a 12.5% or 15% polyacrylamide gel (Fujifilm Wako) and electroblotted onto a polyvinylidene fluoride membrane (Bio-Rad), as previously described.[40] To block nonspecific binding sites, TBS containing 0.1% Tween 20 with 5% nonfat dry milk was used for anti-AKT, anti-MARCKS, and anti-β-actin antibodies, whereas the PhosphoBLOCKER blocking reagent was used for anti-p-MARCKS and anti-p-AKT antibodies. The membrane was incubated overnight at 4°C with Can Get Signal 1 (Toyobo) and primary antibodies raised against human proteins (Cell Signaling Technology), including anti-p-AKT (Ser473; 1:500 dilution; catalog no. 4060),

as mean ± SEM (*p < 0.05, **p < 0.01, ***p < 0.001; n = 3). (D) Western blotting to evaluate p-MARCKS, MARCKS, p-AKT, and AKT protein expression at 72 h post-transfection in DLD-1 or HT-29 cells. All of the proteins were significantly downregulated in the CM-miR-143 group. β-Actin was used as an internal control. (E) Cell viability at 72 h after siRNA-MARCKS transfection (5 nM) in DLD-1 or HT-29 cells. Compared with control siRNA, siRNA-MARCKS 1 and 2 significantly suppressed cell growth in both cell lines. Data are presented as mean ± SEM (***p < 0.001; n = 3). (F) Western blotting to evaluate p-MARCKS, MARCKS, p-AKT, and AKT protein expression at 72 h after transfection in DLD-1 or HT-29 cells. p-MARCKS and MARCKS were downregulated after transfection of siRNA-MARCKS 1 or 2, whereas p-AKT and AKT were not downregulated. β-Actin was used as an internal control. si-MARCKS1, si1; si-MARCKS2, si2.

anti-AKT (1:1,000 dilution; catalog no. 4691), anti-p-MARCKS (Ser152/156; 1:1,000 dilution; catalog no. 2741), anti-MARCKS (1:1,000 dilution; catalog no. 5607), and anti-β-actin (1:1,000 dilution; catalog no. 3700). Next, membranes were washed 3 times with TBS containing 0.1% Tween 20, incubated at 37°C with horseradish peroxidase (HRP)-conjugated anti-mouse (1:10,000 dilution; catalog no. 7076S; Cell Signaling Technology, RRID: AB 330924) or anti-rabbit immunoglobulin G antibody (1:10,000 dilution; catalog no. 7074S; Cell Signaling Technology, RRID: AB 2099233) along with Can Get Signal 2 (Toyobo) as the secondary antibody, and washed 3 more times with TBS containing 0.1% Tween 20. Immunoblots were visualized using an enhanced chemiluminescence detection kit (PerkinElmer). Protein bands were detected using FUSION-FX7 (Vilber Lourmat, Marne-la-Vallée, France).[41] Protein concentrations were verified with an anti-β-actin mouse antibody. The relative expression of each protein *in vivo* was calculated using ImageJ (1.53a, NIH, RRID: SCR 003070).

Dual-luciferase reporter assay

Extensive analysis of the data from TargetScan 8.0 indicated that miR-143-3p targeted and could bind to the 3' UTR of *MARCKS*. A WT-luciferase reporter vector was designed from the 3' UTR of *MARCKS,* including the miR-143 binding site. The MUT-luciferase reporter vector was designed to not match the 3′ UTR of *MARCKS*. DLD-1 cells (1×10^4/well of a 24-well plate) were cotransfected with 30 ng of the luciferase reporter vectors, 7.5 ng Renilla luciferase vector (internal control), and 10 nM control miRNA or 10 nM Am-miR-143. Each vector was transfected with Lipofectamine 2000 (Life Technologies), and each miRNA was transfected with lipofectamine RNAiMAX, following the manufacturer's protocol. Cells were harvested 24 h after transfection. Luciferase activity was assessed using the Dual-Luciferase Reporter Assay System (Promega, Madison, WI).

Analysis of cell viability, protein expression, and mRNA level *in vitro*

The day before transfection, DLD-1 and HT-29 cells were seeded onto 6-well plates at 5×10^4 mL/well. Three miRNAs (control miRNA, Am-miR-143, and CM-miR-143) were used in the experiments. The control was a nonspecific miRNA with a 5'-GUA GGA GUA GUG AAA GGC C-3' sequence. A nonspecific siRNA, as a control, and two *MARCKS* siRNAs were acquired from Life Technologies. The sequences of the *MARCKS* siRNAs were 5′-GAAGAACAA GAAGGAGGCTGGAGAA-3′ and 5′-CCAUACAGAUGGGUCAU GAAAU-3′. Cells were transfected using lipofectamine RNAiMAX according to the manufacturer's protocol. At 72 h posttransfection, viable cell counts were determined using the trypan blue dye exclusion test (Life Technologies). In addition, western blotting was performed to evaluate p-MARCKS, MARCKS, p-AKT, and AKT concentrations. At 48 h posttransfection, qRT-PCR was performed to evaluate *MARCKS* and *GAPDH* mRNA expression levels.

Statistical analysis

Statistical analysis was performed using GraphPad Prism version 9.00 (GraphPad Software, La Jolla, CA; https://www.graphpad.com/scientific-software/prism/, RRID: SCR 002798). Between-group differences were evaluated using the Student's t tests *in vitro* and Mann-Whitney *U* tests *in vivo*. Multiple comparisons *in vitro* were analyzed using Dunnett's test. All of the data were presented as mean ± SEM. Animal survival was evaluated with Kaplan-Meier plots and log rank survival analysis. Significance was set at $p < 0.05$.

DATA AND CODE AVAILABILITY

Data are available via ProteomeXchange with the identifier PXD041937.

SUPPLEMENTAL INFORMATION

Supplemental information can be found online at https://doi.org/10.1016/j.omtn.2023.102079.

ACKNOWLEDGMENTS

This work was supported by a KAKENHI grant from the Japan Society for the Promotion of Science (grant no. 21H02992) and an Osaka Medical and Pharmaceutical University (OMPU) Internal Research Grant (grant number not applicable). We thank Ms. Akiko Miyamoto (Laboratory of General and Gastroenterological Surgery, Osaka Medical and Pharmaceutical University, Takatsuki, Osaka, Japan), Mr. Takaaki Ishizuka (Research Support Center, Instrumentation Division, Osaka Medical and Pharmaceutical University), Mr. Rintaro Oide (Translational Research Program, Osaka Medical and Pharmaceutical University), and Ms. Akiko Kagotani (Translational Research Program, Osaka Medical and Pharmaceutical University) for their technical assistance. We also thank Dr. Takashi Murakami (Saitama Medical University, Faculty of Medicine, Saitama, Japan) for providing the Luc cell lines. Lastly, we thank Editage (www.editage.jp) for their assistance in English-language editing.

AUTHOR CONTRIBUTIONS

Methodology: J.A., K.T., N.S., K.H., and Y.A. Investigation: J.A., N.S., and K.H. Data curation: J.A. and K.T. Formal analysis: J.A. Roles/writing – original draft: J.A. and K.T. Writing – review & editing: N.S., K.H., Y.T., Y.I., K.K., T.T., M.-A.S., S.-W.L., and Y.A. Supervision: S.-W.L. and Y.A. Project administration: K.T.

DECLARATION OF INTERESTS

The authors declare no competing interests.

REFERENCES

1. Lu, T.X., and Rothenberg, M.E. (2018). MicroRNA. J. Allergy Clin. Immunol. *141*, 1202–1207.
2. Yoshikawa, Y., Taniguchi, K., Tsujino, T., Heishima, K., Inamoto, T., Takai, T., Minami, K., Azuma, H., Miyata, K., Hayashi, K., et al. (2019). Anti-cancer effects of a chemically modified miR-143 on bladder cancer by either systemic or intravesical treatment. Mol. Ther. Methods Clin. Dev. *13*, 290–302.
3. Taniguchi, K., Uchiyama, K., and Akao, Y. (2021). PTBP1-targeting microRNAs regulate cancer-specific energy metabolism through the modulation of PKM1/M2 splicing. Cancer Sci. *112*, 41–50.
4. Wang, V., and Wu, W. (2009). MicroRNA-based therapeutics for cancer. BioDrugs *23*, 15–23.

5. Price, C., and Chen, J. (2014). MicroRNAs in cancer biology and therapy: current status and perspectives. Genes Dis. *1*, 53–63.
6. Taniguchi, K., Wada, S.I., Ito, Y., Hayashi, J., Inomata, Y., Lee, S.W., Tanaka, T., Komura, K., Akao, Y., Urata, H., and Uchiyama, K. (2019). α-Aminoisobutyric acid-containing amphipathic helical peptide-cyclic RGD conjugation as a potential drug delivery system for microRNA replacement therapy in vitro. Mol. Pharm. *16*, 4542–4550.
7. Kitade, Y., and Akao, Y. (2010). MicroRNAs and their therapeutic potential for human diseases: microRNAs, miR-143 and -145, function as anti-oncomirs and the application of chemically modified miR-143 as an anti-cancer drug. J. Pharmacol. Sci. *114*, 276–280.
8. Sugito, N., Heishima, K., and Akao, Y. (2022). Chemically modified MIR143-3p exhibited anti-cancer effects by impairing the KRAS network in colorectal cancer cells. Mol. Ther. Nucleic Acids *30*, 49–61.
9. Akao, Y., Nakagawa, Y., Hirata, I., Iio, A., Itoh, T., Kojima, K., Nakashima, R., Kitade, Y., and Naoe, T. (2010). Role of anti-oncomirs miR-143 and -145 in human colorectal tumors. Cancer Gene Ther. *17*, 398–408.
10. Shen, X., and Corey, D.R. (2018). Chemistry, mechanism and clinical status of antisense oligonucleotides and duplex RNAs. Nucleic Acids Res. *46*, 1584–1600.
11. Takai, T., Tsujino, T., Yoshikawa, Y., Inamoto, T., Sugito, N., Kuranaga, Y., Heishima, K., Soga, T., Hayashi, K., Miyata, K., et al. (2019). Synthetic miR-143 exhibited an anti-cancer effect via the downregulation of K-RAS networks of renal cell cancer cells in vitro and in vivo. Mol. Ther. *27*, 1017–1027.
12. Labatut, A.E., and Mattheolabakis, G. (2018). Non-viral based miR delivery and recent developments. Eur. J. Pharm. Biopharm. *128*, 82–90.
13. Hou, X., Zaks, T., Langer, R., and Dong, Y. (2021). Lipid nanoparticles for mRNA delivery. Nat. Rev. Mater. *6*, 1078–1094.
14. Gilleron, J., Paramasivam, P., Zeigerer, A., Querbes, W., Marsico, G., Andree, C., Seifert, S., Amaya, P., Stöter, M., Koteliansky, V., et al. (2015). Identification of siRNA delivery enhancers by a chemical library screen. Nucleic Acids Res. *43*, 7984–8001.
15. Torre, L.A., Bray, F., Siegel, R.L., Ferlay, J., Lortet-Tieulent, J., and Jemal, A. (2015). Global cancer statistics, 2012. CA A Cancer J. Clin. *65*, 87–108.
16. Heald, R.J., and Ryall, R.D. (1986). Recurrence and survival after total mesorectal excision for rectal cancer. Lancet *1*, 1479–1482.
17. den Dulk, M., Krijnen, P., Marijnen, C.A.M., Rutten, H.J., van de Poll-Franse, L.V., Putter, H., Meershoek-Klein Kranenbarg, E., Jansen-Landheer, M.L.E.A., Coebergh, J.W.W., and van de Velde, C.J.H. (2008). Improved overall survival for patients with rectal cancer since 1990: the effects of TME surgery and pre-operative radiotherapy. Eur. J. Cancer *44*, 1710–1716.
18. Fujita, S., Mizusawa, J., Kanemitsu, Y., Ito, M., Kinugasa, Y., Komori, K., Ohue, M., Ota, M., Akazai, Y., Shiozawa, M., et al. (2017). Mesorectal excision with or without lateral lymph node dissection for clinical stage II/III lower rectal cancer (JCOG0212): a multicenter, randomized controlled, noninferiority trial. Ann. Surg. *266*, 201–207.
19. Wanebo, H.J., Koness, R.J., Vezeridis, M.P., Cohen, S.I., and Wrobleski, D.E. (1994). Pelvic resection of recurrent rectal cancer. Ann. Surg. *220*, 586–595.
20. Yamamoto, M., Taniguchi, K., Masubuchi, S., Tominaga, T., Inomata, Y., Miyamoto, A., Ishizuka, T.A., Murakami, T., Osumi, W., Hamamoto, H., et al. (2019). An in vivo mouse model of pelvic recurrence of human colorectal cancer. Sci. Rep. *9*, 19630.
21. Arima, J., Taniguchi, K., Yamamoto, M., Watanabe, T., Suzuki, Y., Hamamoto, H., Inomata, Y., Kashiwagi, H., Kawabata, S., Tanaka, K., et al. (2022). Anti-tumor effect of boron neutron capture therapy in pelvic human colorectal cancer in a mouse model. Biomed. Pharmacother. *154*, 113632.
22. Fong, L.W.R., Yang, D.C., and Chen, C.H. (2017). Myristoylated alanine-rich C kinase substrate (MARCKS): a multirole signaling protein in cancers. Cancer Metastasis Rev. *36*, 737–747.
23. Eguchi, A., De Mollerat Du Jeu, X., Johnson, C.D., Nektaria, A., and Feldstein, A.E. (2016). Liver bid suppression for treatment of fibrosis associated with non-alcoholic steatohepatitis. J. Hepatol. *64*, 699–707.
24. Ding, L., Li, Q., Chakrabarti, J., Munoz, A., Faure-Kumar, E., Ocadiz-Ruiz, R., Razumilava, N., Zhang, G., Hayes, M.H., Sontz, R.A., et al. (2020). MiR130b from Schlafen4(+) MDSCs stimulates epithelial proliferation and correlates with preneoplastic changes prior to gastric cancer. Gut *69*, 1750–1761.
25. Miyamoto, S., Tanaka, T., Hirosuna, K., Nishie, R., Ueda, S., Hashida, S., Terada, S., Konishi, H., Kogata, Y., Taniguchi, K., et al. (2022). Validation of a patient-derived xenograft model for cervical cancer based on genomic and phenotypic characterization. Cancers *14*, 2969.
26. Tseng, W., Leong, X., and Engleman, E. (2007). Orthotopic mouse model of colorectal cancer. J. Vis. Exp. *10*, 484.
27. Heijstek, M.W., Kranenburg, O., and Borel Rinkes, I.H.M. (2005). Mouse models of colorectal cancer and liver metastases. Dig. Surg. *22*, 16–25.
28. Kobaek-Larsen, M., Thorup, I., Diederichsen, A., Fenger, C., and Hoitinga, M.R. (2000). Review of colorectal cancer and its metastases in rodent models: comparative aspects with those in humans. Comp. Med. *50*, 16–26.
29. Wilmanns, C., Fan, D., O'Brian, C.A., Bucana, C.D., and Fidler, I.J. (1992). Orthotopic and ectopic organ environments differentially influence the sensitivity of murine colon carcinoma cells to doxorubicin and 5-fluorouracil. Int. J. Cancer *52*, 98–104.
30. Tokumaru, Y., Tajirika, T., Sugito, N., Kuranaga, Y., Shinohara, H., Tsujino, T., Matsuhashi, N., Futamura, M., Akao, Y., and Yoshida, K. (2019). Synthetic miR-143 inhibits growth of HER2-positive gastric cancer cells by suppressing KRAS networks including DDX6 RNA helicase. Int. J. Mol. Sci. *20*, 1697.
31. Akao, Y., Kumazaki, M., Shinohara, H., Sugito, N., Kuranaga, Y., Tsujino, T., Yoshikawa, Y., and Kitade, Y. (2018). Impairment of K-Ras signaling networks and increased efficacy of epidermal growth factor receptor inhibitors by a novel synthetic miR-143. Cancer Sci. *109*, 1455–1467.
32. Blackshear, P.J. (1993). The MARCKS family of cellular protein kinase C substrates. J. Biol. Chem. *268*, 1501–1504.
33. Liu, J., Chen, S.J., Hsu, S.W., Zhang, J., Li, J.M., Yang, D.C., Gu, S., Pinkerton, K.E., and Chen, C.H. (2021). MARCKS cooperates with NKAP to activate NF-κB signaling in smoke-related lung cancer. Theranostics *11*, 4122–4136.
34. Eustace, N.J., Anderson, J.C., Warram, J.M., Widden, H.N., Pedersen, R.T., Alrefai, H., Patel, Z., Hicks, P.H., Placzek, W.J., Gillespie, G.Y., et al. (2020). A cell-penetrating MARCKS mimetic selectively triggers cytolytic death in glioblastoma. Oncogene *39*, 6961–6974.
35. Liu, H., Su, P., Zhi, L., and Zhao, K. (2017). miR-34c-3p acts as a tumor suppressor gene in osteosarcoma by targeting MARCKS. Mol. Med. Rep. *15*, 1204–1210.
36. Li, T., Li, D., Sha, J., Sun, P., and Huang, Y. (2009). MicroRNA-21 directly targets MARCKS and promotes apoptosis resistance and invasion in prostate cancer cells. Biochem. Biophys. Res. Commun. *383*, 280–285.
37. El Amri, M., Fitzgerald, U., and Schlosser, G. (2018). MARCKS and MARCKS-like proteins in development and regeneration. J. Biomed. Sci. *25*, 43.
38. Chen, C.H., Statt, S., Chiu, C.L., Thai, P., Arif, M., Adler, K.B., and Wu, R. (2014). Targeting myristoylated alanine-rich C kinase substrate phosphorylation site domain in lung cancer. mechanisms and therapeutic implications. Am. J. Respir. Crit. Care Med. *190*, 1127–1138.
39. Beg, M.S., Brenner, A.J., Sachdev, J., Borad, M., Kang, Y.K., Stoudemire, J., Smith, S., Bader, A.G., Kim, S., and Hong, D.S. (2017). Phase I study of MRX34, a liposomal miR-34a mimic, administered twice weekly in patients with advanced solid tumors. Invest. N. Drugs *35*, 180–188.
40. Taniguchi, K., Sugito, N., Shinohara, H., Kuranaga, Y., Inomata, Y., Komura, K., Uchiyama, K., and Akao, Y. (2018). Organ-specific microRNAs (MIR122, 137, and 206) contribute to tissue characteristics and carcinogenesis by regulating pyruvate kinase M1/2 (PKM) expression. Int. J. Mol. Sci. *19*, 1276.
41. Inomata, Y., Oh, J.W., Taniguchi, K., Sugito, N., Kawaguchi, N., Hirokawa, F., Lee, S.W., Akao, Y., Takai, S., Kim, K.P., and Uchiyama, K. (2022). Downregulation of miR-122-5p activates glycolysis via PKM2 in Kupffer cells of rat and mouse models of non-alcoholic steatohepatitis. Int. J. Mol. Sci. *23*, 5230.

OMTN, Volume *34*

Supplemental information

Antitumor effects of chemically modified miR-143 lipoplexes in a mouse model of pelvic colorectal cancer via myristoylated alanine-rich C kinase substrate downregulation

Jun Arima, Kohei Taniguchi, Nobuhiko Sugito, Kazuki Heishima, Yoshihisa Tokumaru, Yosuke Inomata, Kazumasa Komura, Tomohito Tanaka, Masa-Aki Shibata, Sang-Woong Lee, and Yukihiro Akao

Figure S1

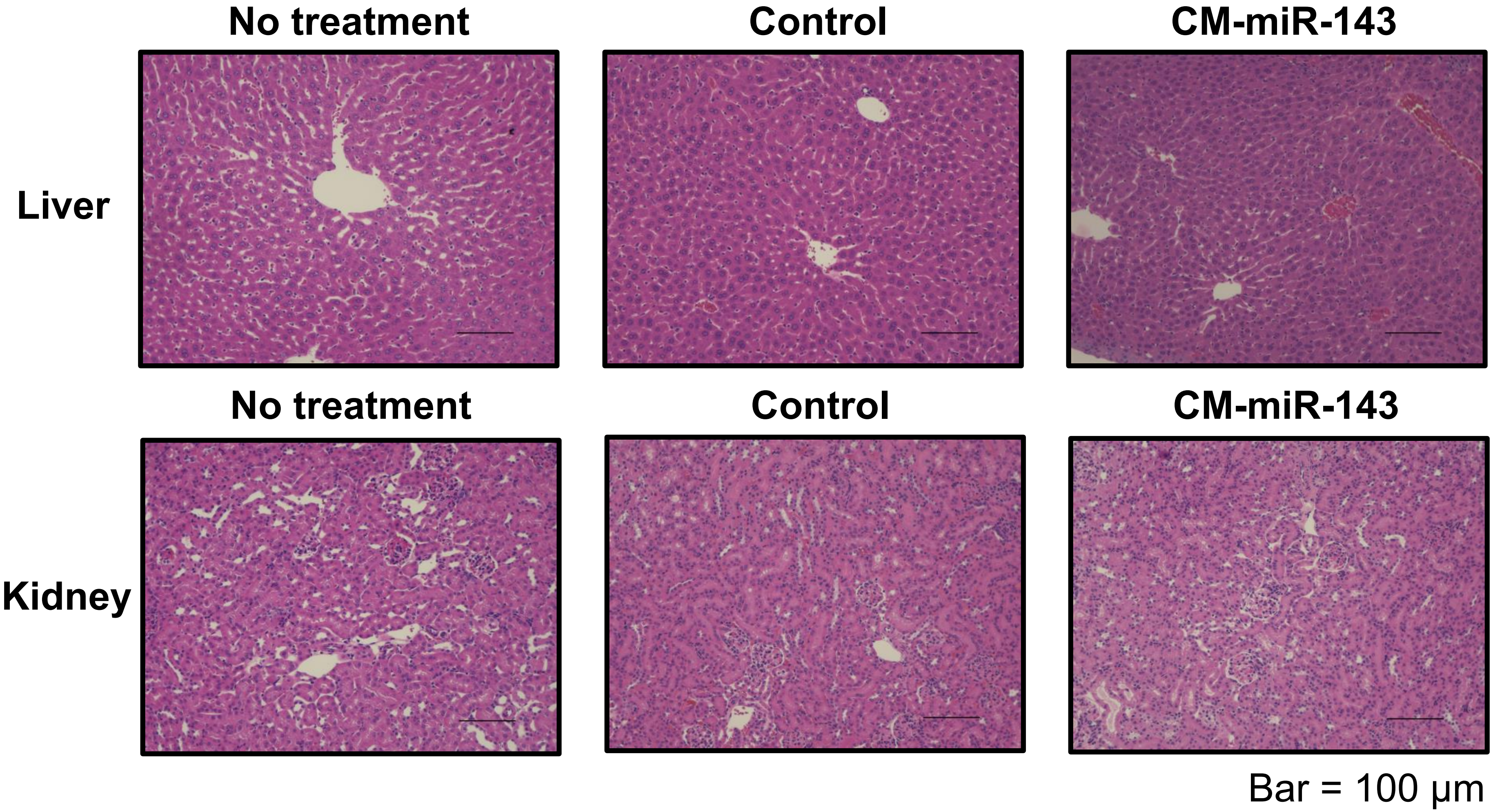

Figure S1. Representative images of pathological liver and kidney.

Liver and kidney images in no treatment, control, and CM-miR-143 groups. In control and CM-miR-143 groups, miRNA lipoplexes were administered at least six times before euthanization. No apparent differences between the liver and kidney were detected across the three groups. No treatment group: mice neither administered miRNA nor Invivofectamine™ 3.0.

Figure S2

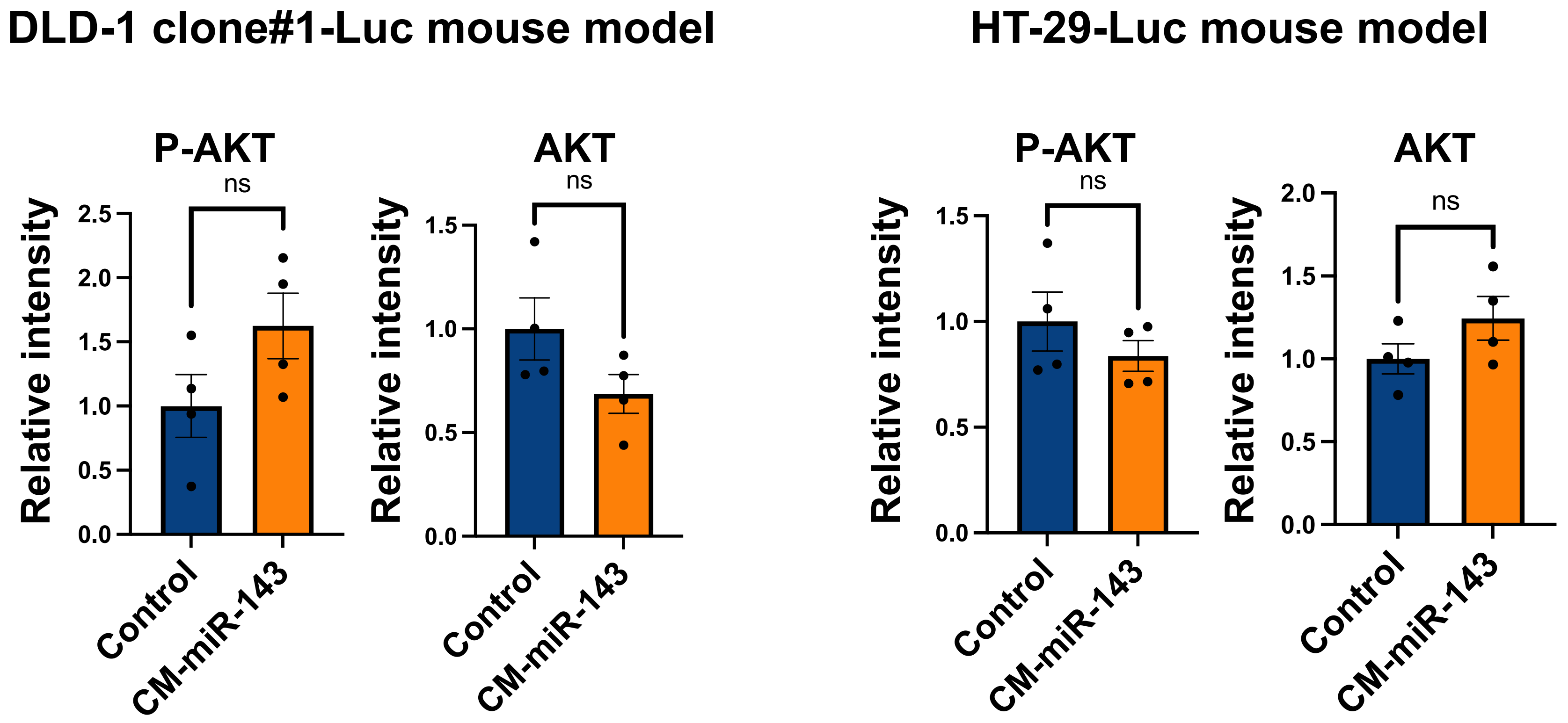

Figure S2. Quantitative intensity of P-AKT and AKT bands.

Quantitative intensity of P-AKT and AKT bands from western blots (Figure 3E) were calculated in ImageJ. Neither protein differed significantly between the control and CM-miR143 groups, based on tumor samples from DLD-1 clone#1-Luc and HT-29-Luc mouse models. Data are presented as mean ± SEM (*$P < 0.05$; $n = 4$). SEM, standard error of the mean.

Figure S3

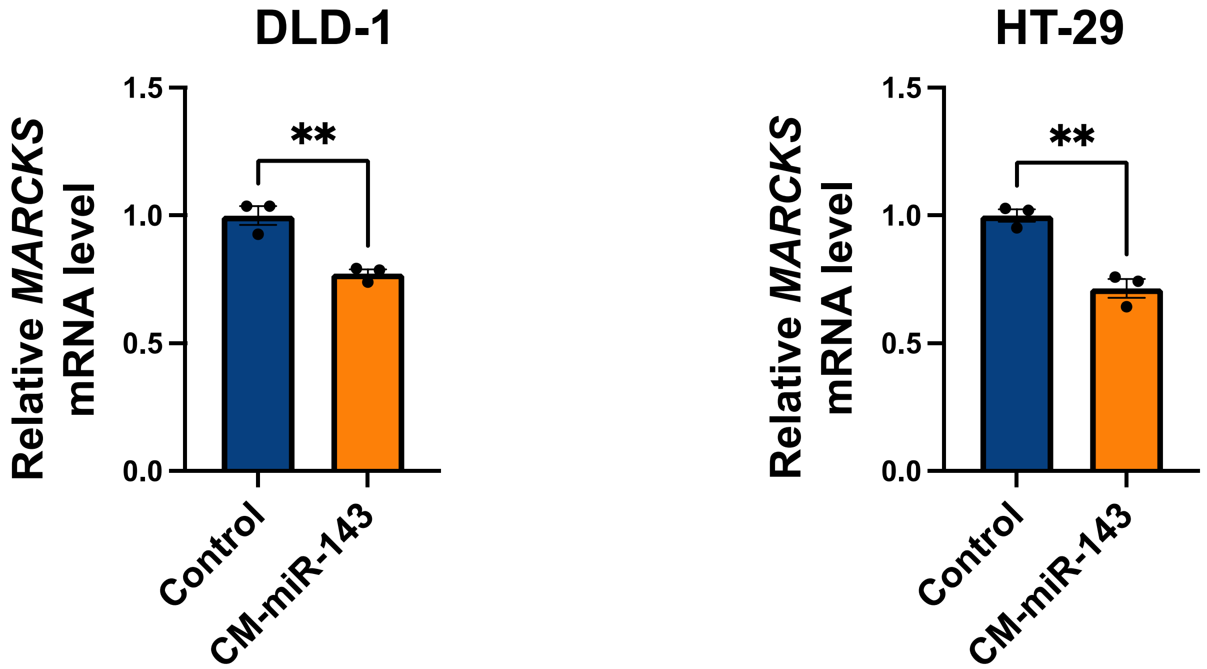

Figure S3. ***MARCKS*** **mRNA expression level**

The level of *MARCKS* mRNA expression was evaluated by qRT-PCR 48 h after transfection with control (10 nM) or CM-miR-143 (10 nM) with lipofectamine RNAiMAX in DLD-1 or HT-29 cells. CM-miR-143 suppressed *MARCKS* mRNA expression compared with the control. *GAPDH* was used as an internal control. Data are presented as mean ± SEM (**P < 0.01; n = 3). SEM, standard error of the mean.

Figure S4

Chemically modified miR-143

S: 3'-GGUCUCUACGUCGUGACGUGGAGU-5'

AS: 5'-U^G^AGAUGAAGCACUGUAGCUCA^dT^dT-3'

N: 2'-Fluoro RNA, N: 2'-O-Methyl RNA, N: RNA without modification,
^: Phosphorothioate, dT: deoxythymidine, N: mismatch

Figure S4. Scheme of CM-miR-143 development.

Red and blue letters indicate modifications with fluorine and methoxy groups in the 2′ position of the sugar ring, respectively. The black letter indicated no modification, "^" indicates phosphorothioate, and dT represents deoxythymidine. S:Sense RNA, AS:Anti-sense RNA

Table S1. Validation of the miR143 and *MARCKS* interaction

miRNA gene name (ID)	miR143 (406935)
Target gene name (ID)	*MARCKS* (4082)[a]
Species name (ID)	*Homo sapiens* (9606)
Sequence of the target region 5′-3′ (searched against TargetScan 8.0)	CAUCUC
Genomic location of MTI (microRNA-target interaction)	113860770-113860777
Genomic location of 3′ UTR[b]	191-197

[a]*MARCKS*: myristoylated alanine-rich C kinase substrate

[b]3′UTR: 3′ untranslated region
